# Associations between nature exposure, screen use, and parent-child relations: a scoping review

**DOI:** 10.1186/s13643-024-02690-2

**Published:** 2024-12-19

**Authors:** Marina Torjinski, Dylan Cliff, Sharon Horwood

**Affiliations:** 1 ARC Centre of Excellence for the Digital Child, Bentley, Australia; 2 School of Psychology, Deakin University, Locked Bag 20000, Geelong, 3220 Australia; 3 School of Education, University of Wollongong, Wollongong, Australia

**Keywords:** Scoping review, Screen use, Parent-child relations, Green space, Nature therapy, Children

## Abstract

**Background:**

Recent research suggests that children spend increasing amounts of time engaging in screen-based activities and less time outdoors in natural environments. There is a growing body of theory-driven literature evidencing that child screen use and exposure to nature are associated with wellbeing outcomes in contrasting ways. However, few studies have explored their combinative effects, and the relational family context has been largely overlooked.

**Objective:**

This scoping review explored associations between early-late childhood nature exposure, screen use, and parent-child relations to identify research gaps and inform future research direction.

**Methods:**

This review was guided by Arksey and O’Malley’s five-stage methodological framework and other relevant guidelines for scoping reviews. A search of five electronic databases (PsycINFO, MEDLINE complete, ERIC, EMBASE, and Cochrane library) was conducted along with additional hand-searches from inception to 9/08/2024. Peer-reviewed articles published in English between 2012 and 2024 were included.

**Results:**

A total of 390 articles were screened by title and abstract and full text review of 96 articles was conducted. Following additional searches (hand-search and reference lists), a total of 23 eligible articles were identified. Evidence is presented in tabular and textual form and described using qualitative thematic analysis. The synthesis revealed that the relevant body of research is novel, heterogenous, and fragmented. There are various pathways through which children’s screen use and engagement with nature interact within the family context; however, research exploring their synchronous and bidirectional effects on relational family processes is limited.

**Conclusion:**

Findings emphasize the importance of investigating children’s screen use and engagement with nature from a relational paradigm. Future studies should explore the mechanisms underpinning the reciprocal influences of nature and screen use on dyadic family processes and relational outcomes across early-late childhood.

**OSF registration:**

https://doi.org/10.17605/OSF.IO/TFZDV.

**Supplementary Information:**

The online version contains supplementary material available at 10.1186/s13643-024-02690-2.

As modern childhood becomes increasingly characterized by an uptake of new, portable, and connected screen-based technologies, children are spending less time outdoors engaging with the natural world [1, 2]. Despite the numerous advantages and opportunities afforded by digital technologies (e.g., access to innovative learning and communication), there is growing evidence that excessive, or problematic screen use is adversely associated with a range of childhood behavioral, emotional [3], psychosocial, and sleep outcomes [4]. Presently, Australian children exceed national screen time recommendations [5, 6] and engage in fewer health-promoting activities known to be protective factors for physical, psychological, and social wellbeing. For example, increases in childhood screen use have corresponded with reductions in time children spend playing, exercising, and socializing in natural environments [7–10]. Interestingly, many of the adverse child health outcomes related to problematic screen use appear to map inversely with the beneficial health outcomes associated with nature exposure. For example, where problematic screen use has been associated with increases in psychological stress [11], time in natural environments has been found to promote cognitive restoration [12, 13] and reductions in both physiological and psychological stress [14]. A small body of literature suggests that through unique restorative effects and psychosocial pathways, nature exposure has the potential to counteract some of the potential adverse health outcomes associated with problematic screen use [15]. However, findings are mixed. The mechanisms underpinning this relationship are poorly understood and family-related factors have not been adequately explored. The question remains as to how the beneficial health effects of nature exposure interact with problematic screen use across childhood, and what role parent-child relations play in this dynamic.

## Problematic child screen use within a family context

Inconsistencies in definitions of problematic child screen use present a challenge to synthesizing the relevant literature [16], and the term is often used interchangeably with phrases such as “excessive screen time” and “screen addiction.” This review will draw on Domoff, Borgan, and Radesky’s [17] definition of childhood problematic media use (screen use) as “excessive patterns of screen use that lead to interferences in daily functioning.” This conceptualization of problematic child screen use is informed by the distinct developmental stages characterizing childhood and the crucial role of caregiver-child interactions during this period. In the context of early-late childhood (0–12 years), problematic screen use may impair self-regulation and manifest in a range of problematic behaviors such as preoccupation with screen-devices, screen-related deception, social withdrawal, or reduced interest in other activities [17]. Such behaviors have the potential to influence children’s psychosocial wellbeing and development through a complex and dynamic interaction of screen-related, child-related, and contextual factors. According to the interactional theory of childhood problematic media use [17], distal factors such parent-child demographics and digital environment design interact with proximal processes such as child temperament [18, 19] and interactional family dynamics [20, 21] to shape maladaptive patterns of child screen use.

Although problematic child screen use is a multi-dimensional construct that interacts with various family-level factors, it is typically operationalized across the literature as a unidimensional measure of total excess screen time. This has significant practical implications. Existing awareness campaigns and interventions are informed by national and global screen time guidelines (e.g., Australian Government Department of Health [5], World Health Organization [22]) that predominantly focus on parents limiting their children’s screen time. For example, parents should not let their children use screens for more than 2 h per day. However, parents often struggle to uphold their ideal screen time limits despite knowledge of potential harms and intention to reduce their child’s screen use [23]. Accordingly, many interventions targeting parental media awareness do not translate into effective screen-limiting practices [24].

## The challenges of screen-time messaging

Existing screen-time messaging often evokes feelings of parental guilt and may corrode parental self-efficacy beliefs [25]. In the family context, parental self-efficacy can be conceptualized as a parent’s beliefs and attitudes about their ability to parent effectively and is significantly related to parenting behaviors [26, 27]. Parents who have higher parental self-efficacy are more likely to engage in effective screen-related family practices, such as upholding healthy screen-time boundaries for their children [28]. Furthermore, many parents are averse to negatively framed screen time messaging and report that positive parent-child interactions and activities have been overlooked by existing parenting campaigns [29]. In a digitally evolving world, where exposure to screen devices is inevitable, there is a need to move beyond inflexible and often unrealistic childhood screen time guidelines towards exploration of positive parenting strategies that may have multiple beneficial and significant effects on children’s screen-related outcomes.

## Individual and relational benefits of nature

Nature exposure or ‘green time’ is receiving growing empirical attention for its health-promoting effects on wellbeing. Outdoor spaces characterized by features of nature (such as forests, beaches, and tree-lined parks) have unique benefits to psychological [30, 31] cognitive [12, 13] and social [32–35] health outcomes for both children and adults. For example, exposure to natural environments has been shown to reduce negative psychological states such as anxiety, lower salivary cortisol concentration (associated with physical and psychological stress), improve cognitive function, increase parasympathetic nervous activity, and lower sympathetic nervous activity (for a review see Yao et al., [36]). Recent studies also demonstrate that beyond physical proximity, nature connectedness has significant benefits to wellbeing [37–39]. The beneficial and wide-ranging effects of human connection to nature have made it a noteworthy topic of investigation across a broad range of research domains, including education, urban planning, environmental psychology, engineering, corporate psychology, medicine, and allied healthcare.

## Contrasting effects of nature and problematic screen use

In context of this review, cross-sectional studies have consistently revealed that nature exposure and problematic screen use independently act on child and adolescent wellbeing in contrasting ways. However, only a small body of literature has explored the combined or reciprocal influences of screen use *and* nature exposure [15]. These studies have typically drawn on Attention Restoration Theory [40] and Stress Reduction Theory [41] to propose that the restorative effects of nature may counter some of the psychosocial processes vulnerable to prolonged screen use. However, the mechanisms underpinning the relationship between child screen use and nature exposure are unclear and the relational family context is underexplored.

## The challenges of unidimensional outdoor measures

Across the broader field of health behavior research, literature investigating the relationship between outdoor time and screen use is largely concentrated around studies of physical activity and sedentary behavior [42]. Evidence consistently demonstrates that time spent indoors is associated with increased sedentary behaviors such as screen time, and time spent outdoors is associated with increased levels of physical activity [43]. Movement behavior studies typically draw on the displacement hypothesis to rationalize that the increasing prevalence and uptake of screen media is the main reason for reduced time children spend on other activities like playing outdoors [44]. These studies adopt measures of “total outdoor time” without investigating the unique influence of different outdoor environments. This is a noteworthy limitation, as exercising in natural environments has been shown to uniquely influence a range of health and wellbeing outcomes (for a review see Brito et al., [45]). For example, compared to walking in urban environments, walks in nature can result in greater reduction of stress and negative affect [46] and produce better results on cognitive tasks [13]. The beneficial effects of exercise and exposure to natural environments are likely to interact in synergistic ways [47]. Hence, associations between screen use, outdoor time, and active lifestyle behaviors should be investigated through a nuanced interactional perspective, beyond the substitution of time from one activity to another.

## Relational family contexts

An important consideration of this review is the paucity of research exploring how nature exposure and problematic screen use interact with parent-child relations (relevant studies have focused primarily on individual rather than inter-personal health outcomes). This gap is particularly salient to the early-mid childhood cohort, where development is characterized by dyadic processes between children and their caregivers—shaping patterns of behavior that are likely to persist through later life [48]. Given that parents play a key role structuring their children’s free time, parental beliefs and attitudes have a significant influence on their children’s play and activity preferences [49]. For example, parent’s safety concerns have consistently been identified as a leading obstacle to children’s outdoor time [49–52] through direct restriction of outdoor activities and transmission of limiting belief systems. Furthermore, children’s direct engagement with natural environments is significantly influenced by parental attitudes towards, and emotional connection with, the natural world [53, 54]. For example, Passmore and colleagues [54] revealed that parental nature connectedness was the strongest predictor of children’s engagement with nature, above and beyond proximity to natural spaces. Other research has demonstrated that family-based nature activities have unique benefits to psychosocial child outcomes above and beyond other leisure-time activities [55]. Such findings emphasize that children’s health behaviors cannot be observed in isolation from parent-child dynamics and other parent-level factors.

## Review objectives

A summary of the literature provides strong evidence for meaningful relationships between screen use, nature exposure, and parenting factors in combinations of conceptual pairs. A triangulation of evidence suggests these variables are interwoven in meaningful ways; however, the interactions between all three key variables have not been fully explored across the literature. The broad nature of this review may shed light on the ways in which screen use and nature exposure interact with family dynamics in the context of early-late childhood and help determine areas where more specific research questions can progress the field. Furthermore, this review will aim to tease out the unique role of nature exposure within broader areas of research, such as studies exploring outdoor movement and sedentary behaviors.

The review objectives are as follows:

- To map the scope of existing literature that explores all three variables of nature exposure, screen use, and parent-child relations across childhood.
- To gather information about how key definitions for screen use, nature exposure, and parent-child relations are conceptualized, defined, and measured across the literature.
- To synthesize findings from a range of literature and identify conceptual and methodological gaps, limitations, and recommendations.
- Inform future research and guide child-health guidelines to generate evidence-based alternatives for parental screen use management.

## Method

### Review framework and development of the research question

The focus of this review was to source research investigating the conceptual overlap of screen use, nature exposure, and parent-child relations in the context of early to late childhood. A priori searches identified that the relevant literature was characteristic of highly heterogenous population samples, variable definitions, study designs, methodologies, and domain-specific theoretical frameworks. Hence, a mixed-method scoping review was adopted to allow for a systematic, multidisciplinary examination across a broad range of literature to map the intersection of key themes and identify knowledge gaps [56]. This review broadly followed Arksey and O’Malley’s five stage framework for scoping reviews [57] with methodological recommendations from Levac and associates [58] and the Joanna Briggs Institute [59]. Reporting adhered to the Preferred Reporting Items for Systematic Reviews and Meta-Analysis Protocols extension for scoping reviews (PRISMA-ScR [60]; [see Additional file 1]). The Population-Concept-Context approach [59] was used to guide the review objectives, research question, definition of key terms, and eligibility criteria. Prior to this review, an a priori scoping review protocol was published [61].

The research question guiding this review is: *What is the scope of existing literature, including construct definitions, methodological limitations and areas for future research, that explores nature exposure, screen use, and parent-child relations across early-mid childhood?*

### Searching the literature

The explorative nature and conceptual novelty of this review necessitated a broad search of research domains, contexts, and geographical localities, and inclusion of wide-ranging study designs and methodologies. Considering the review focus, inclusion of qualitative studies was necessary to adequately capture parental perceptions, attitudes, and processes with respect to family screen use and nature exposure. Early-late childhood (birth-12 years) precedes the developmentally distinct period of adolescence, and is a time when children’s socio-emotional development is shaped by dyadic interactions with their caregivers, whom they are highly dependent on [48]. Publications were limited to the last 12 years to reflect the increasing prevalence and unique influence of novel technologies and emerging media use trends.

Preliminary searches were conducted in PsycINFO using key concept terms in varying combinations to guide development of the initial search strategy. It was important to create a highly sensitive search string to identify publications that may appear misaligned at face value yet contain data relevant to the review focus. A systematic search strategy using keywords and subject terms was developed and independently reviewed by two liaison-librarians and is documented in the review protocol [61]. The original search string was streamlined by replacing searches of thematic pairs with a search of all three key themes together (see Table 3 in Appendix for final search strategy). The following electronic databases were searched from July 2022: PsycINFO, MEDLINE complete, ERIC, EMBASE, and Cochrane library. The first author conducted a supplementary hand-search through Google Scholar and The Children in Nature Network research database (23/11/2022), as well as a backwards and forwards reference list search of all publications identified for inclusion (24/11/2022). A second reviewer screened the additional references against the final inclusion/exclusion criteria and consensus was reached regarding articles suitable for inclusion. All searches were repeated by both reviewers up to August 2024.

### Eligibility criteria

Table 1 outlines the selection criteria for this review, with notations specifying the screening stage at which revisions and adjustments were made.

**Table 1:** Inclusion and exclusion criteria

*Inclusion criteria*	*Exclusion criteria*
***Population***	
*Children:* • Children aged 0–12 • Typically and non-typically developing ***Parents and caregivers:*** • **A biological or non-biological primary legal carer to a child between 0 and 12 years who lives with the child full-time (i.e. more than 5 days a week)**	• Adolescents • Staff, educators
***Concept***	
• ***Articles that cover all three review themes*** • **Themes as either predictor or outcome variables** • **Review themes to appear within study methodology, outcomes or identified themes (for qualitative designs)** Review themes *Nature exposure* • Physical exposure to nature • Proximity to nature • Perceptions or attitudes towards nature including emotional orientation towards nature, nature connectedness, perceived proximity to nature, barriers to access of natural spaces • At title/abstract screening, “outdoors” is acceptable, however article must specify nature in full-text screening *Screen use* • Use of any screen-based technological device, including traditional modes such as television and computers, as well as modern touch-screen devices such as smartphones and tablets *Parent-child relations* • All aspects of parent-child and parent related variables, including parent-child interactions, parenting practices, and parental perceptions	• Time outdoors where features of nature are not explicitly specified (e.g., outdoor basketball court) • Virtual nature engagement (virtual reality, screen projection, screen-based nature games) • Auditory experiences of nature sounds • ^a^No investigation of parent-level factors beyond demographic data • ^b^Studies that do not extend key review themes beyond isolated illustrative examples • ^b^Screen-based app development where screen use theme does not extend beyond specific digital features and applications
***Context***	
• Quantitative, qualitative, or mixed methods study designs (including observational and intervention studies) • All study methodologies (e.g. self-report or objective measures) • Peer reviewed journal articles published in English between 2012–2023 • Any geographical location • Research domains: education, medicine, health-sciences, psychology, urban planning, engineering, computer science	• Gray literature, case studies, reviews, editorials, study protocols, dissertations, poster and conference abstracts, reports, books, opinion papers • Unpublished data • Hospital, clinical, workplace, or childcare setting • ^a^Animal studies, chemistry, ocular pathology, pediatrics, prenatal studies, inpatient studies, career studies • ^a^Studies with exclusively pedagogical or educational outcomes

^a^Added during title/abstract screening stage

^b^Added during full text screening

(a) Nature refers to any outdoor space characterized by features of the natural environment; this may include green spaces such as forests, blue spaces such as beachscapes, or urban outdoor spaces such as tree-lined streets and parks

### Study selection

All articles from the electronic database search were imported to the online software Covidence [62] and duplicates were removed. Titles and abstracts were screened independently by two reviewers through a two-phase iterative review process, as recommended for scoping reviews [58]. The primary aim of the first round of title/abstract screening was to identify methodologically suitable research papers covering all three review themes. During this stage of screening, authors permitted studies investigating “outdoor time” due to the nestling of concepts around nature and the broader outdoor environment. Refinements to the search criteria followed author discussion and included identification of irrelevant research areas and clarification of concept definitions (Table 1). During the second screening round, both reviewers again independently screened titles and abstracts against the refined screening criteria and there were no reviewer conflicts necessitating resolution. Where articles met inclusion/exclusion criteria, or further investigation of article relevance was necessary (for example to determine whether nature exposure was specified or measured as a facet of outdoor time), articles were moved to the next screening stage. Two independent reviewers screened full article texts and discrepancies were resolved through a team discussion which generated some additional refinements to the inclusion criteria (Table 1).

### Charting the data

A customized data charting form was developed prior to the review [61] and collaboratively revised by the authors throughout the extraction process as recommended for scoping reviews [58, 59]. Pertaining to the specific review objectives, information on study variables was extracted only for key review themes. Data from included publications was charted by author 1 (M.T) and cross-checked by author 2 (S.H) using the revised data charting form. Extracted data covered: descriptive information (title, author, year, country of publication), key study aim(s), research domain, concepts of interest, population (characteristics, total number), methodology/analysis, covariates/confounds, relevant findings, limitations, and recommendations. A formal risk of bias assessment was not performed [57].

### Collating, summarizing and reporting the results

In keeping with the central tenets of the scoping review process [57], results were extracted and synthesized in alignment with the key review objectives. First, information within the populated data charting tool was hand coded by author 1 (M.T) and then further developed by identification of overarching themes. Relationships and connections between themes were mapped using mind-mapping software CmapTools [63], and critically discussed by all authors throughout an iterative mapping process. A textual summary of the data was synthesized using qualitative thematic analysis and results reflect the final thematic categories.

## Results

**Fig. 1:**
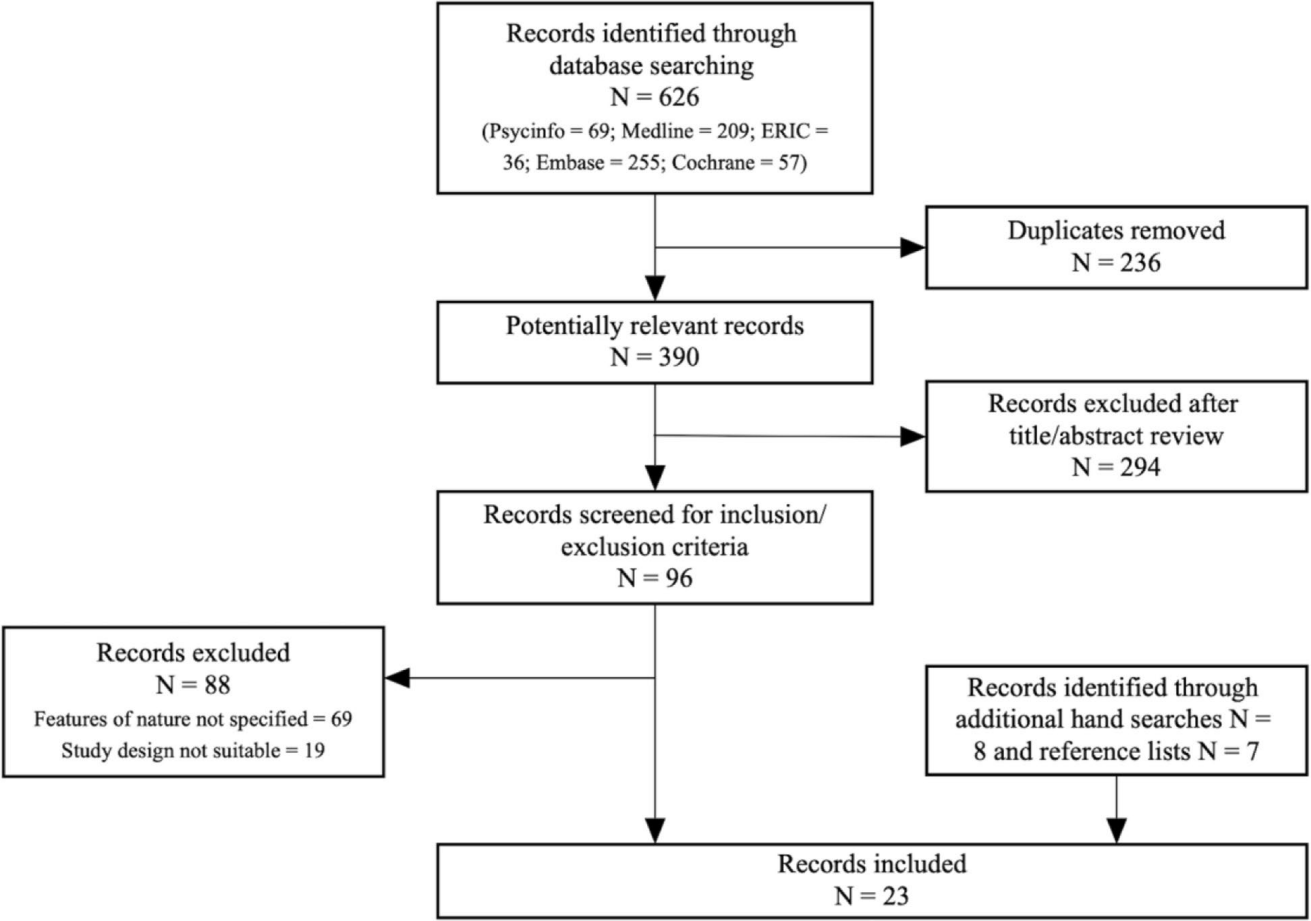
Study search, screening and selection

A total of 626 records were identified following database searches. After removing duplicates (236), 390 articles were screened by title and abstract by two authors (MT, SH). A total of 96 articles were retained for full text review. The screening and selection process is detailed in Fig. 1. After full text review, 8 peer-reviewed journal articles were included from the search of databases and an additional 15 articles were identified through hand searching and reference lists.

### Study characteristics

Articles were distributed over a 12-year period (2012–2024; clustering in 2021) and represented a range of research domains and geographies. Details of eligible study characteristics (including study aims, sampling methods, and relevant constructs) are summarized in Table 2. Publications adopted a range of qualitative (*n* = 7), mixed-methods (*n* = 6), and quantitative (*n* = 10) research approaches involving parent, child, and hybrid samples (10 studies collected both parent and child data with child ages ranging from 3 to 18). Mixed-methods studies incorporated a variety of complimentary data collection methods, including field observations, pre-post program surveys and online questionnaires. Qualitative techniques included interviews, in-App recordings, and focus groups employing various methods of analysis (e.g., thematic analysis, phenomenological approach [64]). One longitudinal study was identified and none of the four eligible intervention studies met experimental conditions [65–68].

**Table 2:** Relevant study characteristics

*Qualitative research papers*					
ID, Domain	Study Characteristics	Primary research aims	Relevant constructs		Relevant results
(Abdullah et al., 2022) [64] Social Sciences, Education	Qualitative; FG interviews, Maldives, children from public schools across 7 island environments: [11, 12] years, 56%♀, *n* = 34	Exploring contextual factors that influence nature experiences among 11-12 year-old children in their local island environments in the Maldives	Children’s nature experiences: children’s perspectives on opportunity/orientation related factors (C-IQs)		• Children’s nature experiences influenced by 4 contextual factors: preferences, opportunities, constraints & freedom • Although many children chose routine screen-based pastimes, nature-based activities were preferred whilst children were in nature places • Perceived constraints (i.e., parental restrictions) deter use of available opportunities, regardless of residential location
			Preference for screen-based activities (results)		
			Influence of family/parents (P-IQs/ results)		
(Ceylan, M. 2018) [69] Social Sciences, Education	Qualitative; interviews, Turkey, parents who attended nature activities [30–59] years 36%♀, *n* = 50 & their children [8–13] years* n* = 70	Identify barriers to engaging parents & children in nature-activities, gather parental perspectives around benefits of nature & examine children’s preferences for electronic or nature-based activities	PS time in nature/ barriers to access nature/perceived benefits of nature on child development (P-IQ)/ preference for nature vs playing with tablet/computer/phone (C-IQ)		• Parents desire to spend more time in nature activities with their children however describe barriers associated with: distance, cost, work fatigue, safety concerns, lack of “nature specialists.” • Positive impacts of nature activities on individual & relational health perceived by parents • Parents perceived influence of nature exposure & ST as contrasting • Children preferred nature activities yet viewed screen use as “compulsory.”
			As above		
			Focus on family nature visits/parent & child views on family nature engagement (results)		
(Kawas et al., 2021) [66] Computer Science	Qualitative; interviews & in-App audio recordings, US, Parents (*n* = 15) & their tween children [8–12] years, 52%♀,* n* = 23 (15 families)	Examining parents’ & tweens’ shared experiences using the “Nature Collections” App (NCApp) in facilitating engagement with outdoor exploration during a tweens transitional stage of technology use	Joint family nature-based explorations during NCApp (objective in-App audio sampling of interactions & parent-reports)		• Key themes: Family experiences of tweens’ App use during nature exploration/ concerns & tensions around tweens use of technology more broadly during transitional developmental period • NCApp engaged parents & tweens in positive shared nature experiences • NCApp provided tweens with opportunity to negotiate autonomy in natural spaces however this was impacted by geographical limitations • Parents reported they would make exceptions to ST limits with technology that facilitates social family interactions & time outdoors promoting NC
			Transitional tech use/ parents’ ST rule		
			Family joint media engagement & experiences of technology: parent & child reflections on family experiences (using the App & generally)		
(Om et al., 2021) [70] Computer Science	Qualitative; interviews/workshops, South Asia, Parents (*n* = 11) adults, 82%♀ & their children (7-8 years,* n* = 12) 67%♀	To investigate existing routines around nature for urban children & explore design of technologies to enhance children’s engagement with nature	P-IQs: perspectives on children’s outdoor activities/children’s workshops: perspectives on outdoor time & preferences around nature		• Children in Bhutan mostly visit nature with their families • Children shared positive views around the social, imaginative, stimulating, interactive & sensory experiences of outdoor play • Technologies have the potential to enhance nature engagement • Screen-based devices can be disruptive to children’s immersive & social experiences in nature
			Technology explored as possible facilitator to children’s nature exposure (aim)		
			Parental perspectives/role of family		
(Rios & Menezes., 2017) [71], Education	Qualitative; Participatory FGs, Portugal, Children from four Portuguese schools [5 – 10 years] 45%♀, *n* = 31	Explore children’s environmental views, including learning about nature, perceived environmental problems & possible solutions	Children’s environmental views/ environmental education		• Experiences with family, community, schools & the media (news & children’s TV programs) were reported sources of learning about nature & environmental awareness shaping children’s environmental concerns • Children’s descriptions of experiences involving pro-environmental behaviors were situated within family contexts
			Learning about nature vicariously through media		
			Family-based experiences & interactions		
(Ruckert et al., 2024) [72], Multidisciplinary	Qualitative; Questionnaire, US, parents of children aged 9 & 10 (82%♀,* n* = 49)	Investigate nature experiences shared between parents & children, with a focus on interactions with animals	Parent-child interactions in nature/ parent-child animal encounters		• Parents reported child/family animal encounters involving digitally mediated vicarious experiences were common, often linked to direct animal encounters, & engaged children’s interests & care for animals • Family experiences with nature provided a range of psychosocial experiences & benefits for parents and children
			Vicarious (digitally mediated) nature experiences		
			Relational family bonding/ experiences involving caregiver influence in child-nature relationships		
(Skinner et al., 2023) [73], Education	Qualitative; Online research instrument, Ireland, mothers [29 – 56 years] of children aged 2 – 19, Gender NP	Investigate the impact of home-schooling on working parents, family & school relationships during the Pandemic lockdown to identify useful parental coping strategies & generate future recommendations	Creativity in nature as a family experience/ family-based outdoor learning		• Parents reported that outdoor learning experiences with their children were therapeutic • Reported benefits to outdoor learning included: exercise, stimulating children’s creative, imaginative & autonomous play, opportunities for learning without screens, as well as connection to/appreciation for nature • Parents reported that children’s preferences for screen-based activities were both challenging to navigate & motivated parents to prioritize outdoor activities
			Parental screen management/ screen related challenges and concerns		
			Parental experiences of home-schooling during the Pandemic lockdown—including perceived challenges, parental roles & family dynamics		
*Mixed-methods research papers*					
ID, Domain	Study Characteristics	Primary Aims	Relevant constructs	Relevant measures	Relevant results
(Griffin et al., 2024) [68], Primary care	Mixed methods; US, pilot intervention study, pre-post surveys: parents of 4—9 year olds (91%♀,* n* = 22) & qualitative interviews with pediatric providers (80%♀, *n* = 4) & parent subset (70%♀, *n* = 10)	To develop & pilot test a primary care-based family-centered behavioral intervention to pro- mote outdoor active play in 4 –10 year old children	Intervention to promote active outdoor nature play/ parental perceptions/ screen-related barriers & family-level facilitators to active outdoor nature play	EV: Parent resources to promote outdoor nature play for children/ age-appropriate nature toy for children OV: Provider & parent perceptions around feasibility, acceptability & preliminary efficacy of program following administration in pediatric clinic/ pre-post parent-child engagement in outdoor PA	• Development (providers): ST as potential barrier/ parental encouragement as facilitator • Feedback (parents): clearer instructions & recommendations for parents, promote outdoor play as alternative for ST to children • No statistically significant pre-post changes in PA and outdoor time
(Hackett et al., 2021) [67] Health Professions, Social Sciences	Mixed methods; pre-post-program survey, US, parent/guardian & child pairs, trios & families from various Milwaukee neighborhoods & surrounds. *n* = 22; youth [8–16] years, adults [18–60] years, 88%♀	Investigating a pilot program to encourage nature-based activity among urban youth & families through environmental education & mentorship	Nature-based outdoor recreation programs/ perceptions around outdoor nature engagement	EV: Joint participation in Nature Mentors programs. OV: Efficacy of programs. Quant: outdoor skills self-efficacy/ weekly program evaluations. Qual IQs: programs features & beliefs around nature engagement	• Perceived barriers included contextual/ environmental factors (e.g., safety & proximity) & attitudes/perceptions (norms, outdoor skills self-efficacy, habitual intentions towards screen-based activities) • Children’s preferences shifted from indoor screen-based to nature-based family activities • Participants perceived program as beneficial for relational wellbeing
			Screen-based activities	Identified within children’s “intention” theme	
			Family-based program /parent & child barriers to nature engagement	Relational program focus/ perceived barriers included contextual & parent-child level factors (i.e., attitudes & norms around nature, outdoor skills self-efficacy)	
(Kaymaz et al., 2017) [74] Medicine	Mixed-methods; parent surveys/ draw & write surveys (children)/field observations in parks & neighborhoods, Turkey, children [6–12] years (*n* = 418) & their parents (17% ♀, *n* = 383)	Investigation into children’s outdoor leisure trends & factors that influence use of urban green spaces in Cayyolu neighborhood of Ankara in Turkey	Factors that influence children’s GS use	EVs: Demographic factors. DVs: PQ: Parental leisure tendencies (including GS/outdoor items) & perceptions around children’s use of outdoor environments. Child surveys: ideal outdoor environments. Field Obs: activity type/duration/engagement	• Interaction was observed between park visiting patterns of parents & children • Perceived benefits of outdoor time, safety concerns & design characteristics affected parents influence on children’s use of GSs • EVs did not influence use of GS • Watching TV was the most preferred leisure-time activity for children • Most children preferred playing with playground equipment when in GS/ parents perceived these facilities as unsatisfactory
			Use of TV &PC	Response options for children’s preferred leisure time activities	
			Parents & children’s outdoor leisure trends/ parental perceptions	Parental perceptions towards children’s use of natural spaces/relationships between parents & children’s outdoor leisure trends	
(Nielsen et al., 2021) [75] Environmental Science, Medicine	Mixed-methods; FG interviews/questionnaire, Denmark, children from 5-8 grade at Danish schools [11–15] years, 59%♀ (interview sample *n* = 34, questionnaire *n* = 1148)	Investigating influence of smartphone use on children’s outdoor experiences	Access & availability of nature/ children’s outdoor experiences	Building density of school/Qs related to nature places & activities/ Children’s perspectives on their outdoor time & preferences around nature	• Children highly dependent on smartphones outdoors/ dependence increases with age • Smartphones can facilitate outdoor experiences through children’s & parents’ sense of safety, children’s social connectedness & opportunities to enhance their outdoor experiences
			Children’s smartphone use	Children’s Qs: children’s dependency on/use of smartphones when outdoors	
			Smartphones to facilitate family contact	Identified theme: smartphone to connect with parents when outdoors	
(Skar et al., 2016) [76] Health Professions, Social Sciences	Mixed-methods; online survey containing open ended Q, Norway, parents of children aged 6 –12 years (children: 48.6%♀), *n* = 3160	To identify & explore barriers for children’s engagement with nearby nature	Children’s use of nearby nature & green areas	Parent-report barriers/facilitators to child use of nearby nature & green areas (prescribed categories/ open ended Q)	• Social barriers (e.g., family schedule, parent attitudes) more salient than physical barriers (e.g., access) to children’s use of nature • Child-related barriers included preference for indoor screen-based activities • Higher barriers perceived for boys/ older children
			Child SU influences outdoor time	Prescribed barrier category (quant)/ identified theme (qual results)	
			Influence of parent-level factors	Parental perceptions: barriers to children’s NE/ parent factors influencing children’s nature engagement	
(Waite et al., 2021) [77]	Mixed methods; quant: survey of local providers of outdoor activities, qual: stakeholder & provider interviews & CYP focus groups, UK, Stakeholders, providers, children from low-income families (FG: [8–20] years, *n* = NP). One FG: CYPs with learning difficulties	Exploration of existing access, barriers & potential facilitators to children & CYP from disadvantaged backgrounds to engage in natural environment activities	Definitions of nature as socio-culturally constructed/outdoor activity providers/CYP participation in natural environment activities	Participants allowed to elicit own meanings for nature concepts/ providers: environmental organizations, community groups, independent outdoor adventure & education providers: types & features of nature engagement programs/ constraints to CYP participation (qual results)	• Programs vary in the ways they facilitated family nature engagement • Definitions of the natural environment shaped by program focus (some providers perceived nature-based programs as opportunity to escape from screen-heavy lifestyle) • Socio-cultural factors identified as obstacles to engagement (e.g., social judgment) • CYP’s willingness to engage in nature-based activities depended on individual/family factors
			Preference for screen-based activities	Identified as CYP barrier to engagement with natural environments	
			Intergenerational/ family influences	Influence of family factors on CYP nature participation (across key themes)	
*Quantitative*					
ID, Domain	Study Characteristics	Primary aims	Relevant Independent Variables	Relevant Outcome(s)	Relevant results
(Aggio et al., 2015) [78] Medicine	Cross-sectional; interviews, Scotland, mother-report child data: Sweep 6 Growing up in Scotland study (children [M = 5.9 years], 49%♀, *n* = 3657)	Investigate the association between child’s screen time & mothers’ perception of distance from home to green/ open spaces (GS)	EV: Mothers’ perceived proximity to GS (walking distance from home/minutes) CV: Parent supervised park/playground visits, frequency of outdoor play (occasions p/wk)	DV: Child use of TV & computers (including laptops & gaming consoles) (weekly frequency & duration)	• Further distance to GS = higher child TV viewing (no association with computer use), worse mental health, less frequent PS park visits, lower general health rating & lower SES • No difference = frequency outdoor play & GS distance • Frequency of PS park visits did not alter association between GS & TV viewing
(Dunton et al., 2012) [79] Medicine, Social Sciences	EMA, US, children in 4—8th grade living in Chino California/ surrounding communities, subgroup from Healthy PLACES trial, [9–13] years, 48%♀, *n* = 97	Using mobile-based Ecological Momentary Assessment (EMA) to describe physical & social contexts of children’s PA behaviors	Current activity (various screen-based response options provided), social company (alone or with parents/ family/ friends), physical location (different outdoor contexts) & contextual factors (e.g., level of vegetation, perceived safety)	Children’s frequency of PA across different social/ environmental contexts	• Only EMA entries reporting PA included in final analysis (indoor sedentary activities not reported) • Children’s PA took place most often with friends & family, or family members only • Age, BMI, income, & ethnic differences in PA contexts were observed • Majority of children’s outdoor PA occurred where children reported higher greenery, no traffic, & felt safe
(Ernst. J., 2018) [65] Arts & Humanities, Business Management & Accounting, Social Sciences	Post-test-only evaluation design; program description matrix & parent/guardian questionnaire, US, parents & guardians from nature play programs (21 zoo & aquarium sites) *n* = 210 (mothers 59.6%, fathers 20.7%, grandparents 7.3%, other 12.4%)	Investigating the influence of various nature-based programs on encouraging family participation in nature-based recreation & overcoming perceived barriers to spending time in nature	EV: Family engagement in nature-play programs (variations across program characteristics: structure, setting, role of parents/ family). PVs/Antecedent OVs: impact of participation on beliefs, attitudes, self-efficacy & strength of barriers (including “difficulty getting my family to unplug from technology”)	OV: Intention towards future family participation in nature-based recreation	• Site-level programs were collectively effective in strengthening motivations towards family engagement in nature-based recreation & decreasing perceived barriers (including technology-related) • Perceptions of program effectiveness & strength of barriers varied based on prior participation in nature-based recreation (program appearing more effective for families who were frequently spending time in nature) • Families who did not already spend frequent time in nature perceived every barrier as significantly stronger
(Keith et al., 2022) [80] Environmental Science, Social Science	Cross-sectional; questionnaire, Australia, CYP from 5-8 grade at Sydney schools [8–14] years, 55%♀ (all CYP *n* = 1037; 8—12 years *n* = 588)	Evaluate predictors of NC & investigate effects of NC & environmental self-identity on urban children’s conservation behaviors	PVs: DNEs (frequency of visits to green & blue spaces/ frequency of nature-focused activities/ participation in outdoor recreation)/ learning about nature (including screen-based learning via television and internet)/ household biodiversity/ local greenness/ population density/ local blueness), NC, environmental self-identity	OV: Conservation behaviors (willingness to conserve nature/ frequency of environmentally responsible behaviors), NC	• NC = large effect on conservation behaviors, mediated by environmental self-identity • Time spent in green & blue spaces = weak direct effect on NC, mediated by engagement with nature-focused activities (strongest predictors of NC alongside frequency of outdoor learning & reading about nature) • Using internet to learn about nature = weakest relationship with NC
(Lu et al., 2020) [81] Health Professions, Medicine	Cross-sectional; questionnaire & ActiGraph accelerometry, China, preschool children in urban area of Tianjin, China, PATH-CC Study, children [3–6] years (all children *n* = 980, Actigraph subgroup *n* = 134) & families (mothers 71%, fathers 21%, grandparents 8%)	Exploring correlates between home & neighborhood environmental characteristics with preschooler’s sedentary behavior (SB) & physical activity (PA)	EVs: Neighborhood characteristics (e.g., distance/ frequency of going to outdoor PA facilities, environmental quality, social support)/ home characteristics (e.g., grandparent as primary caregiver, presence of garden, TV & computers in child’s bedroom/house)/children’s outdoor play/parent-supervised visits to PA facilities	OVs: Sedentary time & PA (objective measure: accelerometry & parent-report questionnaire: daily frequency/duration)	• Children’s outdoor play correlated with lower sedentary time & higher PA/ access to home media equipment was associated with higher levels of leisure-time SB in children/ grandparents as key caregivers correlated with higher sedentary time + SB & lower PA in children • No correlation found between environmental accessibility & outcomes however indirect associations observed between distance to PA facilities & children’s PA outcomes, through increased frequency of parent-supported visits to PA facilities & child’s outdoor play • Social support associated with children spending more time in outdoor play & going to PA facilities more often
(Rosen et al., 2021) [82] Multidisciplinary	Longitudinal; survey, US, children from younger sub-sample (T1 *n* = 68 [53%♀], T2 *n* = 53) & their caregivers	Investigating predictors of child psychopathology during the Pandemic & exploration of protective factors that may buffer against Pandemic related stressors	Protective factors: time spent in natural green spaces (days p/wk), passive ST & news consumption (hrs p/d), family routine & coping strategies (including family support-seeking)/ Pandemic-related stressors: various family-related factors (health, social, financial, home environment)	Internalizing & externalizing psychopathology (child)	• Structured family routine, less passive ST, lower exposure to news media & more time in nature associated with better mental health outcomes in CYP • Children with higher ST showed strong positive associations between pandemic-related stressors & both concurrent/ prospective psychopathology • The strong association between pandemic-related stressors & psychopathology was absent for children with lower ST & news media consumption
(Schoeppe et al., 2016) [83] Medicine	Cross-sectional; questionnaire, Belgium, Greece, Hungary, Germany, Norway, Children (*n* = 3300, [10–12] years, 51%♀) & parents (*n* = 2933, 83%♀), sample = UP4FUN study	Investigate associations between parent/child domain-specific PA & ST activities & determine whether associations are moderated by parent & child gender	PVs: Parent PA & ST variables included: TV & DVD viewing, computer & games console use (min p/d), joint outdoor activities with child (outdoors/outdoors in natural areas/outdoors in other green spaces such as in parks)	OVs: Child PA & ST variables (same as parents, however one measure of total outdoor leisure time)	• Maternal but not paternal modelling of healthy active behaviors (sport participation, outdoor activities & walking for transport) was associated with higher participation in these activities in children • Maternal & paternal modelling of TV, DVD, computer & games consoles was associated with higher screen-based activities in children • The influence of parental modelling appears to be stronger in parent–child pairs of the same gender
(Sheldrake & Reiss., 2023) [84] Science education, Environmental education	Cross sectional; questionnaire, England, Children from primary schools across various regions of England (*n* = 679, [7–10] years, 52.6%♀)	Explore associations between children’s views around nature and different nature-related aspects of education and life	PVs: [5-point Likert responses] Children’s activities & engagement with nature (time in nature, living near nature, watching nature-related media, reading nature-based books, parental encouragement to spend time in nature)/ children’s nature views (same as OVs)	OVs: Appreciation of nature/ affinity towards animals/oneness & responsibility for nature/ interest in learning about nature/ aspirations towards nature-based careers	• Children expressed positive views concerning nature • Parental nature time encouragement = positively predicted children’s nature appreciation/ responsibility • Nature-related media = positively predicted interest in nature-based learning & responsibility for nature • Girls expressed more positive views about nature
(Shyleyko et al., 2023) [85] Medicine, Pediatrics	Cross-sectional; survey, Canada, caregivers of CYPs (*n* = 210, [3–18 years], 48%♀)	To assess activity levels & role of built environment among overweight & obese CYPs referred to a pediatric weight management clinic	PVs: Risk factors for higher ST & lower PA (availability of electronic devices in bedroom/ daily ST/ perceived barriers to child’s PA/ frequency of walking or biking to various locations)	OV: Parental perceptions on children’s PA	• 55% of children had electronic devices in bedroom • Most children exceeded national ST recommendations (males & older children had more ST) • Although most respondents lived within 2 km of GS, it was the least common place for PA • No statistically significant differences for estimates of ST & PA across residential type, number of parents in the household, parental education or overweight parent
(Soga et al., 2018) [51] Environmental Science, Social Sciences	Cross-sectional; questionnaire, Japan, grade 5/6 school children from 45 elementary schools in Tochigi, Japan, *n* = 5801). Gender NP	Investigating associations between frequency of children’s DNEs & NC, family members’ nature orientation, urbanization of school surrounds, time pressure & inclination towards screen media	Family members nature orientation (positive or negative attitudes towards nature-based activities)/ children’s nature relatedness/opportunity (degree of urbanization of school surrounding within 1-km radius of school)/inclination towards screen-based media: child time (min p/d) on various screen devices/ various family-level factors	Children’s DNEs: frequency (last month): visiting natural neighborhood spaces, touching wild plants & observing wildlife	• Frequency of children’s DNEs was significantly positively associated with individual nature relatedness & family members’ nature orientation • Inclination towards screen-based media was significantly positively associated with DNEs (contrary to hypothesis) • Degree of urbanization had significant negative influences on frequency of DNEs • Male children participated in nature-based activities more frequently

*EV* exposure variable, *OV* outcome variable, *PV* predictor variable, *CV* covariate, *C-IQs* child interview questions, *CYP* children & young people, *P-IQs* parent interview questions, *NP* not provided, *SB* sedentary behavior, *PA* physical activity, *DNEs* direct nature experiences, *ST* screen time, *GS* green space, *NC* nature connectedness

### Operationalization of relevant constructs

Mirroring findings from preliminary review searches and consistent with the conceptual and methodological heterogeneities characterizing the broader literature, eligible publications adopted diverse research approaches, and operationalizations of the key review themes (see Table 2). Although methodologically diverse, most publications investigated correlates or predictors of child [51, 64, 70, 74–77, 80, 84] or family [65, 67, 69] engagement with nature with a focus on barriers and facilitators. One ecological momentary assessment [79] and three cross-sectional studies [81, 83, 85] positioned relevant constructs in the context of children’s physical activity and sedentary lifestyle behaviors, and the one longitudinal study focused on youth mental health [82].

Studies explored direct nature experiences (e.g., engagement in nature-based programs) as well as perceptions and attitudes towards nature with relevant measures and outcomes broadly separated into two primary constructs, *opportunity* and *orientation*. Although these constructs were observed throughout the literature, only three publications explicitly drew on them to frame research findings [51, 64, 84]. The first construct, *opportunity*, captured environmental and social factors that may facilitate or impede access and utility of natural resources and spaces (e.g., proximity to nature and available time to play outside [76]). Whilst 4 studies used objective opportunity measures [51, 66, 75, 80], the remainder collected self-report data. The second, *orientation*, was conceptualized as children’s and parents’ connectedness, relatedness, and attitudes towards nature. Integrative measures of nature-related concepts were often adopted, for example frequency, duration, and type of green space use as well as attitudes towards nature [74]. Whilst some studies provided explicit and detailed definitions of nature [64, 71, 72, 77, 80, 82, 84] others provided brief illustrative examples or used concepts around nature and outdoor experiences interchangeably.

Operationalizations of screen use varied between unidimensional measures of device-specific [75] or total screen time [76, 85], composite measures incorporating different media types [51, 81–83], comparisons between screen device types [74, 78, 80], and specific screen-based content (e.g., watching nature-related media [84]) and uses (e.g., using internet to learn about nature [80]). Screen use was commonly provided as a response item for *preferred leisure-time activities* or investigated as a barrier to children’s use of natural spaces either through direct quantitative measures or identified qualitative themes. Three studies investigated screen time as an outcome measure [78, 83, 85] and two publications explored the role of specialized media technologies in the context of facilitating children’s nature experience [66, 70].

### Opportunities and orientations towards nature, parental influences, and children’s free time preferences

Research adopting multifactorial perspectives consistently illustrated that children’s free time preferences and activity choices are influenced by both opportunity and orientation related variables that interact with various proximal child and parent-level factors [51, 74, 76]. Examples of proximal family factors include parental attitudes towards their children’s engagement with nature [51, 65, 67, 74, 76, 77], perceived benefits of nature [65, 69], parental leisure-time trends [74], parental self-efficacy beliefs [65, 67], family rules [64], values [76, 77], and routines [69, 76]. An example of the combinative influences of opportunity and orientation related factors was the cross-sectional study conducted by Soga et al. [51]. This study found that children’s direct nature experiences were positively associated with both their own and their family members nature-orientations and were negatively associated with degree of urbanization of school surrounding. However, many children did not visit neighborhood nature spaces even when they were plentiful (e.g., high density of urban greenness) and close in proximity. Other cross-sectional studies demonstrated that the built environment influences children’s behavior indirectly through parent-level factors such as frequency of family-supervised park visits [78, 81], highlighting the significant role of family contexts in children’s use of available nature.

Parent and child factors were commonly explored as perceived barriers to children’s engagement with nature. Adult-imposed restrictions included logistical constraints such as time pressures and obligations, parenting beliefs and household rules around adult-led activities, and unsupervised child play locations (e.g., that children should not venture into places of nature without a parent). Transference of nature-related limiting beliefs and attitudes such as concerns about child safety (e.g., fear of getting hurt from climbing trees or dangerous animals) was also influential [64, 67, 69, 74, 76, 77]. One study that explored both parent and child-level barriers to children’s engagement with nature found that parents perceived social factors such as parental roles, safety concerns and time pressures as more influential in determining parent-child time in nature than environmental barriers such as access and quality of nearby natural areas [76].

A common child-level barrier to children’s engagement with nature, reported by both children [51, 64, 77] and parents [65, 68, 74, 76], was preference for indoor, screen-based leisure activities. In the study by Skar et al. [76] children’s screen time was perceived as “downgrading” the importance of outdoor time and an open-ended qualitative measure revealed that parents believed screen time limits were important to encouraging children’s engagement with nature. Similarly, Waite [77] illustrated that along with adult-imposed rules, lack of available green spaces, and lack of awareness around benefits of nature, a child’s preference for indoor screen-based activity substantially diminished young people’s intentions to engage with nature.

Several studies revealed that when children were provided with outdoor opportunities, they preferred to play in more naturally diverse outdoor places [64, 77, 79] and often chose activities inspired directly by features of nature such as climbing trees [69] or fishing [64]. Dunton [79] explored the contextual factors of children’s physical activity through real-time momentary assessment and found that most children’s physical outdoor activity occurred in locations where children reported higher levels of greenery, no traffic, felt safe, and were accompanied by friends and family.

Intervention studies with participatory designs affirmed that children’s preferences for either screen-based or nature-based activities are sensitive to available opportunities and the role of caregivers in either facilitating or restricting nature-based opportunities. Aside from promoting positive attitude changes towards family nature experiences, participation in family-based nature programs was able to shift parents’ perceptions around barriers to nature engagement as well as children’s free time preferences [65–67]. For example, after following nature-based programs parents reported an increase in outdoor skills confidence [67], greater motivation to spend time in nature with family, and reduced strength of their own perceived barriers [65]. Correspondingly, through a combination of positive shared experiences and attitude changes, children’s preferences shifted from indoor screen-based activities to outdoor, nature-based and family-centered interests [67]. Contrastingly, one pilot intervention study exploring children’s physical activity and outdoor time found no measurable changes in these outcomes post-intervention [68].

Two studies investigated emergent technologies designed to facilitate family engagement with nature [66, 70]. Although Om and colleagues [70] explored urban children’s outdoor routines with the view of informing digital design to promote child-nature interactions, the study revealed that children also perceive nature-based play as an opportunity to disconnect from technology. It concluded that technology design should support children’s nature play “without the feeling of using technology”. Other research by Kawas [66] explored the use of a digital App to engage tweens and their parents in outdoor experiences. Although parents reported broader family tensions around their tweens’ technology use, the App was able to promote children’s and parents’ motivations and intentions towards future nature-based activities.

### Sociocultural influences and demographic differences

Whilst some studies found SES [76, 78, 82], gender [76, 80, 83–85], age [75, 76, 80, 84, 85], and ethnic [79] differences relating to children’s engagement with nature and use of screen-media, others found no associations [51, 65, 74, 81]. Differences most likely reflect heterogeneities between research aims and study designs, as well as unique sociocultural factors reflecting study location. For example, a publication exploring lifestyle behaviors of children in China found that having grandparents as key caregivers correlated with children spending more time sedentary and less time in physical activity [81]. Lu’s findings largely reflected the Chinese social culture of grandparents as primary caregivers whereas in a European sample (where mothers typically provide primary care), mothers had a greater influence on parent-child modelling of lifestyle behaviors [83].

Research adopting mixed-method designs provided context around how different sociocultural lenses interact with demographic factors to shape the way in which participants perceive, prefer, and engage with routines around nature and screen media. For example, in a study from Norway, parents reported higher barriers to engaging with nearby nature for boys and older children (aged 10–12), with high use of screen-devices and lack of initiative for being outside as key barriers for boys [76]. Contrastingly, girls from the Maldives reported higher barriers to nature experiences due to sociocultural expectations and norms such as family/household responsibilities that reduce available time for outdoor free play. Kaymaz [74] explored how family leisure trends specific to the Turkish sociocultural context influenced children’s activity preferences and use of urban green spaces. Although children aged 6–12 desired to spend more time engaging in nature-based play, adult environmental attitudes reflecting wider cultural trends (such as preference for spending leisure-time in malls and safety concerns) indirectly influenced children’s leisure-time activity choices. A study from the UK [77] explored how young people from disadvantaged backgrounds perceive barriers to use of natural spaces. It provided a nuanced depiction of how transmission of intergenerational and sociocultural normative beliefs can influence young people’s intentions to engage in nature-based and screen-based activities. Whilst parents from minority cultures perceived sedentary child activities as socially appropriate, perceptions of nature-based play were imbued with internalized social judgments around poverty. Although young people preferred “wild” natural environments and described being in nature as “relaxing,” they were deterred by perceptions of social exclusion, low levels of parental engagement, and low confidence in socializing outdoors. The literature collectively illustrates that upstream socio-cultural factors interact with demographic variables such as age and gender to influence family patterns of screen use and engagement with nature.

### Parent–child interaction and relational wellbeing

Concepts around parent-child interactions or relational wellbeing were primarily captured through qualitative themes [66, 67, 69, 71–73, 75]. Participants of intervention studies perceived nature-based programs as either directly beneficial to relational wellbeing [66, 67] or effective in promoting family engagement in nature-based recreation through the reduction of perceived individual and family-level barriers [65]. Although parents commonly reported inclinations towards screen-based activities as a barrier to children’s engagement with nature, parents also noted that their children’s use of mobile phones *during* nature exploration facilitated or maintained parent-child communication, an important component of parent-child relational wellbeing [66, 75]. Through the affordances of portable, connected technologies, young people were able to negotiate personal autonomy whilst maintaining their own and their parents’ sense of safety.

Only a small number of studies explored the reciprocal influences of screen use and nature exposure on parenting dynamics and parent-child relational wellbeing [66, 69, 73, 77]. Parents from one study perceived outdoor family-based nature experiences as conducive for positive relational processes, whilst managing child screen use was described as effortful [73]. Kawas [66] found that despite broader family tensions around their children’s use of screen devices, a tech-based Nature-App was able to facilitate positive shared experiences around nature and family bonding. Interestingly, parents reported that they would make exceptions to screen time limits for technologies that facilitate social family interactions and time outdoors connecting with nature. Waite’s multi-method study [77] added the perspective of nature program providers who described nature-based programs as an opportunity “to escape from their (children’s) usual urban and screen-heavy lifestyles” and connect with family.

### Reported limitations and study recommendations

A range of methodological limitations pertaining to study sample and design were reported by eligible studies. Small sample sizes [65, 67, 68, 75, 77], under-representation of fathers [66, 67, 83], non-random sampling [78], and the range of limitations pertaining to the subjective nature of self-report and proxy measures [65, 70, 78, 81–83, 85] were commonly identified. Limitations around causality [78, 81–83] and study generalizability [51, 66–68, 72, 74, 77, 81, 84, 85] characterized the literature. Recommendations included direct responses to sample limitations and study designs (e.g., inclusion of participants from diverse cultural and socio-economic backgrounds, gender balanced samples [66, 70], and incorporation of objective measures [68, 79, 82, 83]) as well as broader conceptual directions for future research. Emphasis was placed on the importance of studies investigating human-nature interactions adopting multi-disciplinary approaches to research and public health policy (e.g., integration of urban design, child development and health) [51, 74, 81].

Although the child perspective is important to foster children’s enjoyment of nature [64, 76], research exploring children’s engagement with nature should involve parents [74] and primary caregivers [81]. A number of studies suggested that family-centered interventions should focus on dyadic and mutual processes that support health-promoting behaviors for both children and their parents [83], for example, encouraging norms around outdoor family participation and self-efficacy for overcoming family-level barriers to nature participation [67]. Other recommendations included investigating children’s nature experiences using multiple measures (e.g., duration, intensity) [51], age-sensitive refinement of questionnaire items [84], and targeted approaches for families not already engaging in nature-based recreation [65, 77]. Several studies endorsed the utility of technologies in motivating children to engage with nature [70–72, 75, 84]. Rosen’s [82] longitudinal study recommended limiting children’s passive screen use and increasing time in nature as strategies to attenuate the association between life stressors and youth psychopathology.

## Discussion

Through an exploratory scoping review approach, this study sourced, summarized, and synthesized the literature exploring associations between nature exposure, screen use, and parent-child relations in the context of early-late childhood. A total of 23 eligible articles were identified and revealed that the body of research is novel, heterogenous, and fragmented. Although a diversity of research approaches and contexts provides tentative support for meaningful, synchronous, and complex relationships between key review themes, evidence of causality is limited. Nonetheless, this review provides multiple insights into children’s perceptions, experiences, and routines around screen use and nature within a relational family context. The literature exploring nature exposure, screen use, and parent-child relations across early-late childhood is at present limited and this review should be interpreted as a conceptual map and call out for future research. We emphasize the need for research to adopt multi-disciplinary, multifactorial, and relational perspectives of health to understand concurrent trajectories associated with children’s routines around nature and screen use.

### Family influences and children’s engagement with nature

Findings highlight the important role families play in shaping the interaction of opportunity and orientation-related variables that influence children’s engagement with nature. Caregivers play a pivotal role as gatekeepers to children’s leisure-time activities and across diverse ethnic backgrounds, children under 12 mostly spent time in nature with family members or were reliant on their instrumental support for accessing nature-based opportunities. Consistent with other research [53, 54, 86, 87], parental attitudes towards nature-based activities strongly influenced children’s nature orientations, and children described the way transmission of parental beliefs and family values influenced their motivations towards engaging with nature. Parental involvement was key to the success of family-based programs, which, through education, mentorship, teamwork, and social support, were able to positively shift parents’ and children’s attitudes towards future nature-based recreation.

A recent review by Zhang and associates [88] highlighted that “relational dimensions of (green) places” (i.e., the way humans relate to, engage with, and uniquely experience nature) have received less attention across the literature than material measures (such as residential proximity to green spaces), despite robust associations with health-related outcomes. Participatory design studies included in the present review echoed the importance of such measures in understanding patterns of family nature engagement. Programs with the shared view of fostering positive family experiences around nature were effective in promoting attitudinal changes towards nature-based activities for both children and parents, despite differing exposures and designs. Furthermore, positive outcomes were observed despite environmental opportunities remaining constant (e.g., programs utilizing existing neighborhood green spaces and parks). These findings are congruent with research suggesting that parental nature connectedness may be more influential in predicting children’s connection to nature than time spent in nature and neighborhood characteristics [54]. Interestingly, families already engaging in nature-based recreation were more satisfied with program outcomes and experienced a greater reduction across a range of perceived barriers related to access, time constraints, competing child preferences for screen-based devices, safety, and social support. It is likely that the positive shared meanings that families had created through repeated interactions around nature enhanced perceived benefits and confidence in overcoming barriers. Izenstark and Ebata [55] describe such interactions as processes of symbolic meaning-making that are formed over time through shared routines and “ritualized family experiences.” In line with this view, current findings emphasize that social contexts shaping shared symbolic meanings around nature-based experiences should be an important focus for research and intervention.

Consistent with research demonstrating that family-based nature activities promote positive family functioning in unique ways due to the psychologically restorative effect of natural environments [55], children perceived time in nature as beneficial to wellbeing through mental restoration and positive shared experiences [67, 69]. They described an underlying sense of freedom, relaxation, and escape afforded by characteristics of the natural environment [64, 67, 77]. Children’s preferences for more naturally diverse environments [64, 77, 79] reflects the theoretical supposition that opportunities for immersive engagement with nature are more likely to promote cognitive restoration [40].

Heterogeneities between study aims, designs, and methodologies limited the ability to draw conclusion about dose-related aspects of nature exposure. Nonetheless, this review generated meaningful insights around the importance of perceived nature connectedness and a deeper understanding of how the social contexts of nature-based experiences influence child outcomes. Methodologies that capture attitudes and perceptions around family nature engagement affirm the significance of relational measures of nature in understanding why families do (or do not) engage with available nature and how benefits are derived.

### Nature-based interventions and children’s screen use preferences

The theme of children’s screen use was most frequently contextualized within explorations of barriers to children’s engagement with nature and was captured through both pre-determined response items [51, 74, 76] and qualitative themes [64, 67–69, 73, 76, 77]. However, there was also evidence that family interventions resulting in attitudinal changes around nature could lead to shifts in children’s perceptions around their screen use, including the intention to replace habitual screen-based hobbies with outdoor activities [65, 67]. These shifts hinged upon a new-found or invigorated affinity for nature, increased confidence to participate in outdoor activities, changes in perceived social norms, and increased parental support around nature-based activities. Current findings complement the suggestion that a temporary disconnect from screen-based activities can lead to perceived increases in young people’s connection to nature [89].

### Theoretical perspectives: relational family processes, problematic child screen use, and attention restoration

Although modern technologies provide an unprecedented array of opportunities for children to learn and connect, problematic screen use is a distinct construct that is associated with marked differences across a range of individuals, and relational child health outcomes [4, 90]. Evidence of problematic child screen use was observed in Rosen’s study [82], where strong associations between pandemic-related stressors and psychopathology were only present among children with higher amounts of screen time and news media consumption. However, in this study and across the review literature, the relational dimensions associated with children’s problematic screen use were not explored beyond illustrative examples.

In terms of causal relationships, no study directly investigated whether an increase in family-based nature experiences can influence relational family processes and outcomes associated with children’s screen use patterns and behaviors. However, when framed by relevant theoretical models [17, 40, 55], the literature collectively illustrates various pathways through which engagement with nature may influence relational processes and dynamics implicated in problematic child screen use.

#### Dyadic processes

The interactional theory of childhood problematic media use [17] suggests that certain dyadic parent-child interactions can perpetuate patterns of problematic child screen use. For example, feedback loops between children’s screen-related oppositional behavior, parental stress and low parental self-efficacy towards screen-limiting practices may perpetuate children’s problematic screen use [91]. Whilst rewarding features of some screen-based activities can provide positive reinforcement for children, parents may experience negative reinforcement when difficult child behaviors are temporarily abated through device-led child occupation [17]. Such processes can interfere with the ability for parent-child dyads to learn co-regulation when difficult behaviors emerge—perpetuating maladaptive family patterns associated with problematic screen use. Consistent with other experimental research [92–94], our findings suggest that family nature participation can provide opportunities for joint-family activities that are mutually enjoyable, are mentally restorative, and promote family cohesion through important dyadic processes such as responsiveness and communication. There are several specific pathways through which family nature participation can improve parent-child relationship quality. For example, natural environments that promote a sense of relaxation and cognitive restoration may provide optimal opportunities for children to develop self-regulation skills [95–98] which are associated with positive dyadic family interactions [55]. Despite the evidence supporting these theoretical pathways, the combinative influence of relational processes involved in problematic child screen use and family-based nature engagement on relational outcomes has not been investigated empirically.

#### Parental self-efficacy

Positive shared experiences around nature also led to increases in domain-specific parental self-efficacy [65, 67]. Nature-based parental self-efficacy scales have demonstrated significant positive relationships with measures of nature connectedness and general parental self-efficacy [99]. General parental self-efficacy can predict a parent’s ability to overcome a range of parenting challenges and is associated with healthy family functioning, parent and child health outcomes, and relational wellbeing (for a review see Albanese et al., [100]). To the best of our knowledge, whether increases in parental self-efficacy resultant from positive family engagement in nature can influence media-specific parental self-efficacy has not been empirically tested. However, this is a worthy pursuit given that low levels of both general and media-specific parental self-efficacy have been identified as proximal factors associated with problematic child screen use [17].

#### Social contexts

Social influences such as screen-based peer activities and norms are important factors involved in the maintenance of problematic child screen use [17]. Current findings illustrate that the preferred leisure-time activities of friends and norms around online social interactions were influential to children’s habitual screen use patterns and preferences for screen-based activities over nature-based interests. Both objective [79] and child-report measures [64, 70, 76] illustrated that children preferred to be in places of nature with friends. However, lack of peer interest in nature and social connections revolving around screen-based activities were perceived barriers to engagement with nature [65, 69, 76, 77]. Interestingly, following nature-based programs, children and parents reported that sharing positive experiences around nature with other families contributed to changes in perceived norms around outdoor family activities [65, 67]. Forming new social connections around nature-based activities facilitated a sense of social support and enhanced motivations to engage in future nature participation. Such findings provide compelling examples of how programs designed to engage families in nature-based activities have the potential to indirectly influence children’s screen use habits and preferences through a combination of social and family-level pathways.

#### Technologies to enhance family engagement with nature

A comprehensive examination of specialized technologies designed to promote family nature engagement was beyond the scope of this review (only studies exploring broader themes around children’s screen-use met eligibility criteria). However, the current review shed light on how various technologies and their uses may interact with broader family dynamics around children’s screen use and children’s nature experiences. Findings demonstrated that whilst socially reinforced screen use habits and inclinations can shift children’s preferences away from health-promoting nature-based and family-centered activities, screen-based technologies have the potential to promote positive family experiences around nature through shared enjoyment or learning [66, 70–72, 84]. Depending on specific technological features and uses, research approaches, and explanatory theoretical frameworks, the influence of technologies can be both adversarial and conducive to children’s engagement with nature.

When considering the interactional systems involved in children’s problematic screen use [17], technology that promotes positive family-based nature experiences can provide opportunities to enhance both individual wellbeing and strengthen processes involved in healthy family functioning. However, from the perspective of Attention Restoration Theory [40], there are caveats to the benefits of family-based nature interactions facilitated by screen-based media. To enhance cognitive restoration, nature-based activities should promote a sense of reprieve from the daily distractions, preoccupations, and stressors that induce attentional fatigue. In one study, although a specialized Nature-App was able to promote positive family experiences in nature, themes relating to broader family tensions and apprehensions around children’s screen use emerged among parent-child negotiations specifically related to the Nature-App [66]. Considering that children are often accompanied by parents in places of nature, such findings raise questions about how the relational nuances around children’s use of technology during family-based nature experiences influence the restorative potential of natural settings. Despite the theoretical relevance of Attention Restoration Theory [40] to children’s routines around nature and screen use, and existing evidence suggesting that use of technology in natural settings can disrupt attention restoration [101], eligible studies did not investigate findings from this theoretical perspective. However, based on children’s and parent’s accounts of family nature experiences, suggestions were made regarding development of digital design that can support children’s social interactions through “nature play” whilst minimizing the interruptive influence of technology [70]. Findings are in line with research exploring the influence of digital design on children’s outdoor play experiences [102]. At present, unanswered questions remain about the influence of screen-based technologies on immersive experiences of nature in the family context, for example, whether parental perceptions around children’s use of technology in nature vary between device types, features, and applications. These are important reflections, considering the recent proliferation of technologies designed to engage children with nature.

#### Demographic factors and sociocultural influences

Children’s patterns of behavior and preferences around screen use and nature are dynamic processes that are shaped by their social ecologies which predominantly consist of family systems that interact with distinctive cultural influences. Findings from the study by Skar [76] provide a robust example of how sociocultural norms and family factors can influence gender differences and age-related patterns in children’s screen use and nature exposure. In this study, the competing influence of screen-based activity preferences on children’s engagement with nature increased as a function of age, with higher barriers perceived for boys. Authors posited that Norwegian boy’s tendency to play more screen-based games than girls, influenced their engagement with nature both directly and indirectly though reductions in motivation and lack of social support for engaging in outdoor activities. Concurrently, higher barriers for older children (aged 10–12) were ascribed to the reduced level of parental supervision and facilitation (of nature-visits) during a transitional stage of development characterized by increasing child autonomy. The inverse associations and age-related trends in children’s screen use and nature connectedness are in line with other studies [1, 89, 103, 104]). It is likely that these inverse and age-related trends reflect dynamic interactions between changing parent-roles and increasing autonomy throughout childhood, the growing prevalence and popularity of screen-based activities, and the changing landscape of technological devices and applications targeted at young consumers.

Sociocultural influences were pertinent in shaping attitudes towards nature and screen time as well as children’s leisure-time behaviors. The following two examples illustrate the importance of understanding how sociocultural factors can shape screen time and nature experiences. Contrary to their hypothesis, Soga [51] found that children’s inclination towards screen-based media was positively associated with visits to neighborhood natural places and nature relatedness. This pattern may have reflected the influence of nature-based technologies like Pokemon GO, which are popular with children in Japan. Through specific design features and shared social interest, such technologies can facilitate both connection to natural environments and community [105]. From this perspective, it is likely that findings from Soga’s study reflect the mediating role of specific sociocultural influences.

By investigating the views of individuals from disadvantaged and minority groups, Waite and associates [77] demonstrated that beyond economic factors limiting opportunities for families to access places of nature, intergenerational beliefs interwoven with cultural histories, discourses, and norms can shape the way ethnic groups conceptualize different leisure-time activities. For example, whilst certain ethnic groups associated nature-based play with perceptions of social judgment and exclusion, indoor sedentary activities were perceived as “socially acceptable.” These descriptions illustrate how socio-culturally embedded identities around leisure-time activities can shape related parental beliefs and children’s free time preferences. This study highlights that efforts to engage minority and disadvantaged groups with neighborhood green spaces and nature-based programs should adopt socio-culturally sensitive approaches to messaging, informed by research adopting participatory designs. As well as shifting limiting intergenerational narratives, a focus on socially inclusive ways of promoting nature connectedness can help overcome some of the barriers and inequities inherent to environmental opportunity-related characteristics (such as proximity, access, and quality of green spaces).

### Strengths and limitations

Understanding children’s perspectives is crucial to family-centered research and this review was able to gather meaningful information about children’s perceptions and experiences around nature and screen use. This review also drew together findings from a range of research domains, to provide multi-dimensional perspectives around children’s health behaviors within the family context and provide a multi-disciplinary mapping of areas for future research.

Although a previous review explored associations between children’s screen time and green time [15], only quantitative studies with a focus on individual psychological outcomes were included. The current review used an exploratory population-concept-context framework [59] with a mixed-methods approach to allow for new perspectives to emerge through investigation of relationships and potential pathways between key review themes. The inclusion of qualitative and mixed-methods studies (which provided rich data around both parent and child perspectives and attitudes) generated particularly salient findings. Whilst some studies were limited by lack of objective screen-time data (which provide the most precise representations of child screen use), research exploring interactions between perception and behavior was enriched by methodologies incorporating subjective measures or qualitative investigations of child screen use. Likewise, methodologies capturing the influence of family attitudes and perceptions around children’s engagement with nature were able to provide meaningful insights in response to the current review objectives.

Eligible studies explored the mechanisms underlying parent and child engagement with nature, such as role modelling of behaviors and attitudes towards nature-based activities. However, little emphasis was placed on children’s screen use contexts (aside from studies explicitly investigating technologies to facilitate nature engagement). Although the literature provides a glimpse into the influence of screen-based preferences on children’s leisure-time activity choices, the influence of media types and uses remains largely unexplored in this context. Data collected for different device types was generally collapsed into single measures of total screen use for analysis [51, 81–83]—a limitation mirroring the broader research investigating children’s screen time and green time [15, 32]. This is problematic as modern devices and digital applications are likely to influence children’s behaviors and play preferences in novel and unique ways [106–108]. Although children’s inclinations towards screen-based activities were perceived as barriers to their engagement with nature, it remains unclear whether certain device types and activities are perceived as greater barriers over others. This is an important limitation, considering the role of technological design in the development of problematic child screen use [17, 108]. Furthermore, central theories expounding the relationship between screen time and nature (Attention Restoration Theory [40] and Stress Reduction Theory [41]) focus on cognitive and psychological processes that may be sensitive to the unique effects of modern screen devices.

Another limitation that may have impacted our review pertains to research pooling variables of interest with other measures or omitting them from analysis. Although methodological decisions were developed in apt response to specific research aims, in the context of this review some opportunities for meaningful comparisons between key variables were missed. For example, screen time data in one study was collected but combined with other variables into a composite measure of leisure-time sedentary behavior [81] whilst another study collected real-time data on children’s screen-activity contexts but did not report findings [79]. Schoeppe and associates [83] measured both parent-child co-visitation to natural spaces and child screen time yet did not investigate and report the associations.

Several papers collected data on outdoor environments with and without features of nature, providing an opportunity to tease apart the influence of these two distinct variables on outcome measures. However, most of these studies collapsed data into unidimensional measures of total outdoor time (e.g., Lu et al., [81])—as commonly seen across the broader literature [44]. One study that explored this distinction [82] provides compelling evidence for the unique influence of nature on child wellbeing (total time outdoors was unrelated to child psychopathology whilst time in nature was associated with better mental health outcomes). Such findings demonstrate that a unidimensional measure of total outdoor time may not capture important distinctions between outdoor exposures and their unique influence on individual and relational outcomes.

Finally, it is important to note that only papers in English were eligible and therefore other relevant publications may not have been included. Resultingly, data relating to and reflecting a range of unique cultural samples may have been excluded from this review.

## Future directions and conclusion

This review contributes nuanced perspectives to the broader health-behavior literature that typically situates children’s screen use and outdoor time in the context of sedentary and active lifestyle behaviors. Through a specific focus on family engagement with natural environments, our findings illuminate some of the complex mechanisms underlying this time-use relationship and highlight the importance of investigating children’s health behaviors from a relational paradigm.

Considering the unique and significant influence of nature on individual and relational wellbeing, future studies should focus on teasing out the conceptual and semantic overlap between nature and the outdoors. Clear definitions and operationalizations will allow for more precise and meaningful within- and between-study comparisons of these two environmental exposures to better inform future research directions, recommendations, and ensuing program developments. Likewise, future research should explore the multi-dimensional facets of screen use (such as the influence of device types and uses) in the context of children’s free time preferences, behaviors, and family dynamics to generate practicable solutions for parents.

Socio-culturally reinforced screen use habits can influence children’s outdoor time through shifts in preferences away from health promoting activities. Consequently, children are less motivated to initiate and engage in outdoor play. However, depending on how screen use is conceptualized, and which outcomes are explored, screen-based technologies can act as both barriers *and* facilitators to children’s engagement with nature. Conversely, participation in family-based nature experiences may influence children’s screen use patterns indirectly through the combinative influences of parental nature orientations, changes in family routines and positive shared experiences on children’s free-time preferences.

Collectively, patterns across findings provide insight into the different pathways through which family-based nature engagement can influence the interactional systems involved in problematic child screen use. Congruent with Izenstark and Ebata’s [55] interactional theory, studies with intervention designs demonstrated that positive family experiences in nature can enhance perceived wellbeing, shift children’s habitual leisure-time preferences, increase parental self-efficacy and provide opportunities to develop positive family interactions. Although problematic child screen use can interfere with processes central to healthy family functioning [17, 90, 91], there was a lack of eligible studies framing children’s screen use from a relational perspective. Future studies should explore the differential impacts and mechanisms underpinning the reciprocal influences of problematic child screen use and family-based nature experiences on dyadic parent-child processes, interactions, and outcomes. For example, studies with qualitative designs should explore how family routines around nature can influence maladaptive child behaviors and family interactions associated with problematic child screen use. Research with longitudinal or experimental designs and larger samples is needed to investigate whether changes in family nature engagement can influence family dynamics directly associated with problematic screen use (such as frequent requests for screen-devices, emotional dysregulation, parental stress, and screen-time self-efficacy).

Research specifically investigating technology designed to promote children’s engagement with nature should consider the differential influence of media types and features on nature-based family dynamics as well as individual processes involved in nature-induced attention restoration. A balanced view is necessary to understand the potential for technology to promote and inspire family engagement with nature whilst optimizing the restorative potential of natural settings.

Digital technologies are fast becoming an inextricable part of daily family life and despite parental concerns around the potential health impacts of problematic child screen use [109], reducing children’s screen time has proved a difficult prospect. Beyond providing alternatives to screen-based activities, efforts to support parents in managing problematic child screen use should focus on promotion of both positive screen uses and health-promoting family routines and interventions that may have direct and indirect impacts on screen-related child behaviors. Such strategies should focus on strengthening the parent-child relationship through improved individual and dyadic wellbeing outcomes and empower parents with the confidence to shape healthy family routines. Parent-child engagement with natural environments offers rich opportunities for positive family routines that are likely to be agreeable for young people [75] and mutually beneficial for parents and their children. Promotion of family engagement in nature-based activities may provide opportunities not only to displace children’s sedentary screen time through shifts in parental attitudes and children’s free time preferences, but to counteract some of the adverse psychosocial outcomes associated with problematic screen use. Importantly, variables related to attitudes around nature such as self-efficacy towards nature-based activities and nature relatedness are amendable, therefore a promising target for intervention.

## Supplementary Information

Additional file 1. PRISMA-ScR (Preferred Reporting Items for Systematic reviews and Meta-Analyses extension for Scoping Reviews) Checklist.

## Data Availability

The datasets generated and/or analyzed during the current study are available in the OSF repository: DOI: 10.17605/OSF.IO/TFZDV.

## Appendix

**Table 3:** Search strategy

**Database**	**Search strategy**	**Comments**
***APA Psycinfo (*****Via***** EBSCO****)*	S1 (TI (green OR nature OR forest OR outdoor OR wilderness) N3 (time OR space* OR exposure OR area* OR playground* OR environment* OR exercise* OR based OR play* OR school* OR experience* OR therap* OR bathing OR learning OR education OR immersion OR connectedness)) OR (AB (green OR nature OR forest OR outdoor OR wilderness) N3 (time OR space* OR exposure OR area* OR playground* OR environment* OR exercise* OR based OR play* OR school* OR experience* OR therap* OR bathing OR learning OR education OR immersion OR connectedness)) S2 TI greenspace* OR AB greenspace* OR TI "blue space*” OR AB “blue space*” OR TI “shinrin yoku” OR AB “shinrin yoku” OR TI “eco-therap*" OR AB “eco-therap*” S3 S1 OR S2 S4 (TI(screen OR screens OR “electronic device*” OR computer* OR hand-held OR media OR tablet* OR mobile-device* OR mobile-phone* OR television* OR I-pad* OR iPad* OR touch-pad* OR touchpad* OR cell-phone* OR smart-phone* OR smartphone* OR I-phone* OR iPhone*) N3 (use OR exposure OR time OR behavio#r* OR addict*)) OR (AB(screen OR screens OR “electronic device*” OR computer* OR hand-held OR media OR tablet* OR mobile-device* OR mobile-phone* OR television* OR I-pad* OR iPad* OR touch-pad* OR touchpad* OR cell-phone* OR smart-phone* OR smartphone* OR I-phone* OR iPhone*) N3 (use OR exposure OR time OR behavio#r* OR addict*)) S5 DE “digital gaming” OR DE “screen time” OR DE “smartphone use” S6 S4 OR S5 S7 (TI (family OR families OR parent* OR mother* OR father* OR “parent-child” OR carer OR caregiver* OR guardian* OR mum* OR mom* OR dad* OR childrearing OR “child rearing” OR maternal OR paternal)) OR (AB (family OR families OR parent* OR mother* OR father* OR “parent-child” OR carer OR caregiver* OR guardian* OR mum* OR mom* OR dad* OR childrearing OR “child rearing” OR maternal OR paternal)) S8 DE "Family" OR DE "Family Relations" OR DE "Family and Parenting Measures" OR DE "Family Conflict" OR DE "Family Relations" OR DE "Child Discipline" OR DE "Childrearing Practices" OR DE "Parent Child Relations" OR DE "Parental Role" OR DE "Parent Child Communication" OR DE "Parental Attitudes" OR DE "Parent Report" OR DE "Parental Attitudes" OR DE "Parental Expectations" OR DE "Parental Characteristics" OR DE "Parental Role" OR DE "Parenting Style" OR DE "Parental Involvement" OR DE “family therapy” OR DE “family intervention” OR DE “mother-child relations” OR DE “father-child relations” S9 S7 OR S8 S10 S3 AND S6 AND S9	Limiters: Peer reviewed 2012-2023
***MEDLINE complete (*****Via***** EBSCO)***	Search strategy as above in *APA Psycinfo (*Via* EBSCO)*	Add into subject headings: (MH "Screen Time") OR (MH "Computers, Handheld + ") OR (MH "Smartphone") OR (MH "Internet Addiction Disorder") OR (MH "Technology Addiction + ") OR (MH "Internet Use") OR (MH "Television + ") OR (MH "Video Games + ") OR (MH "Digital Technology") (MH "Family Relations + ") OR (MH "Parent-Child Relations + ") OR (MH "Maternal Behavior + ") OR (MH "Paternal Behavior") OR (MH "Parents + ") OR (MH "Parenting") OR (MH "Family + ") OR (MH "Family Conflict") OR (MH "Family Characteristics + ") OR (MH "Family Health") OR (MH "Child Rearing + ") OR (MH "Mother-Child Relations + ") OR (MH "Mothers + ") OR (MH "Father-Child Relations") OR (MH "Fathers + ")
***ERIC (*****Via***** EBSCO)***	Search strategy as above in *APA Psycinfo (*Via* EBSCO)*	Add into subject headings: DE "Outdoor Education" DE "Handheld Devices" OR DE "Computers" OR DE "Computer Games" OR DE "Computer Use" OR DE "Internet" OR DE "Laptop Computers" OR DE "Television" OR DE "Television Viewing" OR DE "Video Games" DE "Family (Sociological Unit)" OR DE "Parents" OR DE "Family Attitudes" DE "Caregiver Child Relationship" OR DE "Family Life" OR DE "Parent Child Relationship" OR DE "Parenting Skills" OR DE "Parenting Styles" OR “child rearing”
***EMBASE***	S1: ((green OR nature OR forest OR outdoor OR wilderness) NEXT/3 (time OR space* OR exposure OR area* OR playground* OR environment* OR exercise* OR based OR play* OR school* OR experience* OR therap* OR bathing OR learning OR education OR immersion OR connectedness)):ti,ab S2: greenspace* OR 'blue space*' OR 'shinrin yoku' OR 'eco-therap*':ti,ab S3: 'green space'/exp OR 'forest bathing'/exp S4: #1 OR #2 OR #3 S5: ((screen OR screens OR 'electronic device*' OR computer* OR 'hand held' OR media OR tablet* OR 'mobile device*' OR 'mobile phone*' OR television* OR 'i pad*' OR ipad* OR 'touch pad*' OR touchpad* OR 'cell phone*' OR 'smart phone*' OR smartphone* OR 'i phone*' OR iphone*) NEAR/3 (use OR exposure OR time OR behavio?r* OR addict*)):ti,ab S6: 'screen time'/de OR 'computer addiction'/exp S7: #5 OR #6 S8: family OR families OR parent* OR mother* OR father* OR 'parent-child' OR carer* OR caregiver* OR guardian* OR mum* OR mom* OR dad* OR childrearing OR 'child rearing' OR maternal OR paternal:ti,ab S9: 'child parent relation'/exp OR 'parental behavior'/exp OR 'family dynamics'/exp S10: #8 OR #9 S11: #4 AND #7 AND #10	*• Do not have mapping options selected (use field codes instead)*
*CENTRAL (Cochrane)*	S1: ((green OR nature OR forest OR outdoor OR wilderness) NEXT/3 (time OR space* OR exposure OR area* OR playground* OR environment* OR exercise* OR based OR play* OR school* OR experience* OR therap* OR bathing OR learning OR education OR immersion OR connectedness)):ti,ab S2: (greenspace*) OR (blue NEXT space*) OR (shinrin NEXT yoku) OR (eco-therap*):ti,ab S3: #1 OR #2 S4: ((screen OR screens OR 'electronic device*' OR computer* OR 'hand held' OR media OR tablet* OR 'mobile device*' OR 'mobile phone*' OR television* OR 'i pad*' OR ipad* OR 'touch pad*' OR touchpad* OR 'cell phone*' OR 'smart phone*' OR smartphone* OR 'i phone*' OR iphone*) NEAR/3 (use OR exposure OR time OR behavio?r* OR addict*)):ti,ab S5: Mesh Headings S6: #4 OR # 5 S7: (family OR families OR parent* OR mother* OR father* OR "parent-child" OR carer* OR caregiver* OR guardian* OR mum* OR mom* OR dad* OR childrearing OR "child rearing" OR maternal OR paternal):ti,ab S8: Mesh Headings S9: #7 OR #8 S10: #3 AND #6 AND #9	*Use Medline MESH terms* (MH "Screen Time") OR (MH "Computers, Handheld + ") OR (MH "Smartphone") OR (MH "Internet Addiction Disorder") OR (MH "Technology Addiction + ") OR (MH "Internet Use") OR (MH "Television + ") OR (MH "Video Games + ") OR (MH "Digital Technology") (MH "Family Relations + ") OR (MH "Parent–Child Relations + ") OR (MH "Maternal Behavior + ") OR (MH "Paternal Behavior") OR (MH "Parents + ") OR (MH "Parenting") OR (MH "Family + ") OR (MH "Family Conflict") OR (MH "Family Characteristics + ") OR (MH "Family Health") OR (MH "Child Rearing + ") OR (MH "Mother-Child Relations + ") OR (MH "Mothers + ") OR (MH "Father-Child Relations") OR (MH "Fathers + ")

## References

[1] Larson LR, Szczytko R, Bowers EP, Stephens LE, Stevenson KT, Floyd MF. Outdoor time, screen time, and connection to nature: Troubling trends among rural youth? Environ Behav. 2019;51(8):966–91.

[2] Edwards RC, Larson BMH. When screens replace backyards: strategies to connect digital-media-oriented young people to nature. Environ Educ Res. 2020;26(7):950–68.

[3] Li C, Cheng G, Sha T, Cheng W, Yan Y. The relationships between screen use and health indicators among infants, toddlers, and preschoolers: a meta-analysis and systematic review. Int J Environ Res Public Health. 2020;17(19):7324. 33036443 10.3390/ijerph17197324 PMC7579161

[4] Mallawaarachchi SR, Anglim J, Hooley M, Horwood S. Associations of smartphone and tablet use in early childhood with psychosocial, cognitive and sleep factors: a systematic review and meta-analysis. Early Childhood Res Q. 2022;60:13–33.

[5] Health AGdo. Australia’s physical activity and sedentary behaviour guidelines. Move and Play Every Day; National Physical Activity Recommendations for Children 0-5 Years. 2021.

[6] Watson A, Dumuid D, Maher C, Olds T. Associations between meeting 24-hour movement guidelines and academic achievement in Australian primary school-aged children. J Sport Health Sci. 2022;11(4):521–9. 33359235 10.1016/j.jshs.2020.12.004 PMC9338336

[7] Larson LR, Cordell HK, Betz CJ, Green GT. Children’s time outdoors: results from a national survey. 2011.

[8] Kellert SR, Case DJ, Escher D, Witter DJ, Mikels-Carrasco J, Seng PT. Disconnection and Recommendation for Reconnection. Nat Am Natl Rep. 2017:1-364.

[9] Arnas YA, Deniz ŞS. An investigation of pre-school children’s and their parents’ outdoor play experiences. Pegem J Educ Instruction. 2020;10(2):373–98.

[10] England N. Childhood and nature: a survey on changing relationships with nature across generations, report to natural England: Natural England; 2009.

[11] Sohn SY, Rees P, Wildridge B, Kalk NJ, Carter B. Prevalence of problematic smartphone usage and associated mental health outcomes amongst children and young people: a systematic review, meta-analysis and GRADE of the evidence. BMC Psychiatry. 2019;19(1):1–10. 31779637 10.1186/s12888-019-2350-x PMC6883663

[12] Stevenson MP, Schilhab T, Bentsen P. Attention Restoration Theory II: a systematic review to clarify attention processes affected by exposure to natural environments. J Toxicol Environ Health B Crit Rev. 2018;21(4):227–68. 30130463 10.1080/10937404.2018.1505571

[13] Stevenson MP, Bentsen P, Dewhurst R, Schilhab T. Cognitive restoration in children following exposure to nature: evidence from the attention network task and mobile eye tracking. Front Psychol. 2019;10:42. 30804825 10.3389/fpsyg.2019.00042 PMC6370667

[14] Jones R, Tarter R, Ross AM. Greenspace interventions, stress and cortisol: a scoping review. Int J Environ Res Public Health. 2021;18(6):2802. 33801917 10.3390/ijerph18062802 PMC8001092

[15] Oswald TK, Rumbold AR, Kedzior SGE, Moore VM. Psychological impacts of “screen time” and “green time” for children and adolescents: a systematic scoping review. PLoS One. 2020;15(9):e0237725. 32886665 10.1371/journal.pone.0237725 PMC7473739

[16] Kaur N, Gupta M, Malhi P, Grover S. Screen time in under-five children. Indian Pediatr. 2019;56(9):773–88. 31638012

[17] Domoff SE, Borgen AL, Radesky JS. Interactional theory of childhood problematic media use. Hum Behav Emerg Technol. 2020;2(4):343–53. 36381426 10.1002/hbe2.217 PMC9645666

[18] Clifford S, Doane LD, Grimm KJ, Lemery-Chalfant K, Breitenstein R. Effortful control moderates the relation between electronic-media use and objective sleep indicators in childhood. Psychol Sci. 2020;31(7):822–34. 32558622 10.1177/0956797620919432 PMC7492726

[19] Pace U, D’Urso G, Zappulla C. Internalizing problems as a mediator in the relationship between low effortful control and internet abuse in adolescence: a three-wave longitudinal study. Comput Hum Behav. 2019;92:47–54.

[20] Coyne SM, Rogers A, Shawcroft J, Reschke P, Barr R, Davis EJ, et al. Meltdowns and media: Moment-to-moment fluctuations in young children’s media use transitions and the role of children’s mood states. Comput Hum Behav. 2022;136:107360.

[21] Zhao J, Zhang Y, Jiang F, Ip P, Ho FKW, Zhang Y, et al. Excessive screen time and psychosocial well-being: the mediating role of body mass index, sleep duration, and parent-child interaction. J Pediatr. 2018;202:157–62. 30100232 10.1016/j.jpeds.2018.06.029

[22] Organization WH. Guidelines on physical activity, sedentary behaviour and sleep for children under 5 years of age: World Health Organization; 2019. 31091057

[23] Horwood S, Anglim J, Mallawaarachchi SR. Problematic smartphone use in a large nationally representative sample: age, reporting biases, and technology concerns. Comput Hum Behav. 2021;122:275–81.

[24] Krafft H, Boehm K, Schwarz S, Eichinger M, Büssing A, Martin D. Media awareness and screen time reduction in children, youth or families: a systematic literature review. Child Psychiatr Hum Dev. 2023;54(3):815–25. 10.1007/s10578-021-01281-9 PMC10140095 34855040

[25] Mallawaarachchi SR, Hooley M, Sutherland-Smith W, Horwood S. “You’re damned if you do, you’re damned if you don’t”: a qualitative exploration of parent motives for provision of mobile screen devices in early childhood. BMC Public Health. 2022;22(1):1–13. 36324121 10.1186/s12889-022-14459-0 PMC9629764

[26] Bandura Aa. Self-efficacy: the exercise of control: W.H. Freeman. 1997.

[27] Bandura A. Self-efficacy: toward a unifying theory of behavioral change. Psychol Rev. 1977;84(2):191–215. 847061 10.1037//0033-295x.84.2.191

[28] Thompson DA, Tschann JM. Update on screen-related parenting practices in early childhood. Acad Pediatr. 2020;20(8):1066–8. 32653689 10.1016/j.acap.2020.07.007 PMC7655564

[29] Marsh S, Taylor R, Galland B, Gerritsen S, Parag V, Maddison R. Results of the 3 Pillars Study (3PS), a relationship-based programme targeting parent-child interactions, healthy lifestyle behaviours, and the home environment in parents of preschool-aged children: a pilot randomised controlled trial. PLoS One. 2020;15(9):e0238977. 32941530 10.1371/journal.pone.0238977 PMC7498059

[30] Reyes-Riveros R, Altamirano A, De La Barrera F, Rozas-Vásquez D, Vieli L, Meli P. Linking public urban green spaces and human well-being: a systematic review. Urban Forest Urban Green. 2021;61:127105.

[31] Stier-Jarmer M, Throner V, Kirschneck M, Immich G, Frisch D, Schuh A. The psychological and physical effects of forests on human health: a systematic review of systematic reviews and meta-analyses. Inter J Environ Res Public Health. 2021;18(4):1770. 10.3390/ijerph18041770 PMC7918603 33670337

[32] Mygind L, Kurtzhals M, Nowell C, Melby PS, Stevenson MP, Nieuwenhuijsen M, et al. Landscapes of becoming social: a systematic review of evidence for associations and pathways between interactions with nature and socioemotional development in children. Environ Int. 2021;146:106238. 33189991 10.1016/j.envint.2020.106238

[33] Putra IGNE, Astell-Burt T, Cliff DP, Vella SA, Feng X. Association between green space quality and prosocial behaviour: a 10-year multilevel longitudinal analysis of Australian children. Environ Res. 2021;196:110334. 33075353 10.1016/j.envres.2020.110334

[34] Putra IGNE, Astell-Burt T, Cliff DP, Vella SA, Feng X. Association between caregiver perceived green space quality and the development of prosocial behaviour from childhood to adolescence: latent class trajectory and multilevel longitudinal analyses of Australian children over 10 years. J Environ Psychol. 2021;74:101579.

[35] Putra IGNE, Thomas A-B, Dylan PC, Stewart AV, EmeEseme J, Xiaoqi F. The relationship between green space and prosocial behaviour among children and adolescents: a systematic review. Front Psychol. 2020;11:859. 32425867 10.3389/fpsyg.2020.00859 PMC7203527

[36] Yao W, Zhang X, Gong Q. The effect of exposure to the natural environment on stress reduction: a meta-analysis. Urban Forest Urban Green. 2021;57:126932.

[37] Martin L, White MP, Hunt A, Richardson M, Pahl S, Burt J. Nature contact, nature connectedness and associations with health, wellbeing and pro-environmental behaviours. J Environ Psychol. 2020;68:101389.

[38] Richardson M, Passmore H-A, Lumber R, Thomas R, Hunt A. Moments, not minutes: the nature-wellbeing relationship. Int J Wellbeing. 2021;11(1).

[39] Richardson M, Hamlin I. Nature engagement for human and nature’s well-being during the Corona pandemic. J Public Ment Health. 2021;20(2):83–93.

[40] Kaplan R, Kaplan S. The experience of nature: a psychological perspective. New York, NY: Cambridge University Press; 1989.

[41] Ulrich RS, Simons RF, Losito BD, Fiorito E, Miles MA, Zelson M. Stress recovery during exposure to natural and urban environments. J Environ Psychol. 1991;11(3):201–30.

[42] Gray C, Gibbons R, Larouche R, Sandseter EBH, Bienenstock A, Brussoni M, et al. What is the relationship between outdoor time and physical activity, sedentary behaviour, and physical fitness in children? A systematic review. Int J Environ Res Public Health. 2015;12(6):6455–74. 26062039 10.3390/ijerph120606455 PMC4483711

[43] Pereira JR, Cliff DP, Sousa-Sá E, Zhang Z, Santos R. Prevalence of objectively measured sedentary behavior in early years: systematic review and meta-analysis. Scand J Med Sci Sports. 2019;29(3):308–28. 30456827 10.1111/sms.13339

[44] Casey G, Rebecca G, Richard L, Ellen Beate Hansen S, Adam B, Mariana B, et al. What is the relationship between outdoor time and physical activity, sedentary behaviour, and physical fitness in children? A systematic review. Int J Environ Res Public Health. 2015;12(6):6455–74. 26062039 10.3390/ijerph120606455 PMC4483711

[45] Brito HS, Carraça EV, Palmeira AnL, Ferreira JP, Vleck V, Araújo D. Benefits to performance and well-being of nature-based exercise: a critical systematic review and meta-analysis. Environ Sci Technol. 2022;56(1):62–77. 34919375 10.1021/acs.est.1c05151

[46] Sara LW, Katherine NI, Melissa RM. Walking for well-being: are group walks in certain types of natural environments better for well-being than group walks in urban environments? Int J Environ Res Public Health. 2013;10(11):5603–28. 24173142 10.3390/ijerph10115603 PMC3863862

[47] Thompson Coon J, Boddy K, Stein K, Whear R, Barton J, Depledge MH. Does participating in physical activity in outdoor natural environments have a greater effect on physical and mental wellbeing than physical activity indoors? A systematic review. Environ Sci Technol. 2011;45(5):1761–72. 21291246 10.1021/es102947t

[48] Bronfenbrenner U. Ecological systems theory Six theories of child development: Revised formulations and current issues. Londres: Jessica Kingsley; 1992. p. 187–249.

[49] Wiseman N, Harris N, Downes M. Preschool children’s preferences for sedentary activity relates to parent’s restrictive rules around active outdoor play. BMC Public Health. 2019;19(1):946. 31307424 10.1186/s12889-019-7235-x PMC6631440

[50] Truong MV, Nakabayashi M, Hosaka T. How to encourage parents to let children play in nature: factors affecting parental perception of children’s nature play. Urban Forest Urban Green. 2022;69:127497.

[51] Soga M, Yamanoi T, Tsuchiya K, Koyanagi TF, Kanai T. What are the drivers of and barriers to children’s direct experiences of nature? Landsc Urban Plan. 2018;180:114–20.

[52] Hinkley T, McCann JR. Mothers’ and father’s perceptions of the risks and benefits of screen time and physical activity during early childhood: a qualitative study. BMC Public Health. 2018;18(1):1271. 30453927 10.1186/s12889-018-6199-6 PMC6245522

[53] Barrable A, Booth D. Nature connection in early childhood: a quantitative cross-sectional study. Sustainability (Switzerland). 2020;12(1):1–15.

[54] Passmore HA, Martin L, Richardson M, White M, Hunt A, Pahl S. parental/guardians’ connection to nature better predicts children’s nature connectedness than visits or area-level characteristics. Ecopsychology. 2021;13(2):103–13.

[55] Izenstark D, Ebata AT. Theorizing family-based nature activities and family functioning: the integration of attention restoration theory with a family routines and rituals perspective. J Fam Theory Rev. 2016;8(2):137–53.

[56] Munn Z, Peters MD, Stern C, Tufanaru C, McArthur A, Aromataris E. Systematic review or scoping review? Guidance for authors when choosing between a systematic or scoping review approach. BMC Med Res Methodol. 2018;18:1–7. 30453902 10.1186/s12874-018-0611-x PMC6245623

[57] Arksey H, O’Malley L. Scoping studies: towards a methodological framework. Int J Soc Res Methodol Theory Pract. 2005;8(1):19–32.

[58] Levac D, Colquhoun H, O’Brien KK. Scoping studies: advancing the methodology. Implement Sci. 2010;5(1):69. 20854677 10.1186/1748-5908-5-69 PMC2954944

[59] Peters MDJ, Godfrey CM, Khalil H, McInerney P, Parker D, Soares CB. Guidance for conducting systematic scoping reviews. Int J Evid Based Healthc. 2015;13(3):141–6. 26134548 10.1097/XEB.0000000000000050

[60] Tricco AC, Lillie E, Zarin W, O’Brien KK, Colquhoun H, Levac D, et al. PRISMA Extension for Scoping Reviews (PRISMA-ScR): Checklist and Explanation. Ann Intern Med. 2018;169(7):467–73. 30178033 10.7326/M18-0850

[61] Torjinski M, Horwood S. Associations between nature exposure, screen use, and parent–child relations: a scoping review protocol. Syst Rev. 2023;12(1):217. 37974236 10.1186/s13643-023-02367-2 PMC10652600

[62] Covidence systematic review software. Melbourne (Australia): Veritas Health Innovation; 2022. Available from: https://www.covidence.org.

[63] CmapTools. IHMC Concept Map Software: a knowledge construction toolkit. 2003.

[64] Abdullah F, Ishak NA, Ahmad MZ. Unpacking Determinants of Middle-School Children’s Direct Nature Experiences (DNEs): An Island Perspective. Int J Learn Teach Educ Res. 2022;21(10):19–49.

[65] Ernst J. Zoos’ and aquariums’ impact and influence on connecting families to nature: an evaluation of the Nature Play Begins at Your Zoo & Aquarium program. Visit Stud. 2018;21(2):232–59.

[66] Kawas S, Kuhn NS, Sorstokke K, Bascom E, Hiniker A, Davis K. When screen time isn’t screen time: tensions and needs between tweens and their parents during nature-based exploration. In: Proceedings of the 2021 CHI Conference on Human Factors in Computing Systems; 2021. p. 1–14.

[67] Hackett KA, Ziegler MC, Olson JA, Bizub J, Stolley M, Szabo A, et al. Nature mentors: a program to encourage outdoor activity and nature engagement among urban youth and families. J Adventure Educ Outdoor Learn. 2021;21(1):35–52.

[68] Griffin GM, Nieto C, Senturia K, Brown M, Garrett K, Nguyen E, et al. Project nature: promoting outdoor physical activity in children via primary care. BMC Prim Care. 2024;25(1):68. 38395776 10.1186/s12875-024-02297-5 PMC10885514

[69] Ceylan M. With parental eye, factors that prevent to attend nature activites. Educ Res Rev. 2018;13(24):769–76.

[70] Om C, Brereton M, Dema T, Ploderer B. Design opportunities to enhance children's engagement with nature in Bhutan: A working field theory. In: Proceedings of the 33rd Australian Conference on Human-Computer Interaction; 2021. p. 51–61.

[71] Rios C, Menezes I. ‘I saw a magical garden with flowers that people could not damage!’: children’s visions of nature and of learning about nature in and out of school. Environ Educ Res. 2017;23(10):1402–13.

[72] Ruckert JH, Moreno C, Postigo M, Thurston MJ. Encountering animals cultivates meaningful shared experiences between children and parents. Anthrozoös. 2024:1-22.

[73] Skinner B, Hou H, Taggart S, Abbott L. Working parents’ experiences of home-schooling during school closures in northern ireland: what lessons can be learnt? Irish Educa Stud. 2023;42(3):339–58.

[74] Kaymaz I, Oguz D, Cengiz-Hergul OC. Factors influencing children’s use of urban green spaces. Indoor Built Environ. 2019;28(4):520–32.

[75] Nielsen JV, Arvidsen J. Left to their own devices? A mixed methods study exploring the impacts of smartphone use on children’ outdoor experiences. Int J Environ Res Public Health. 2021;18(6):3115. 33803517 10.3390/ijerph18063115 PMC8002890

[76] Skar M, Wold LC, Gundersen V, O’Brien L. Why do children not play in nearby nature? Results from a Norwegian survey. J Adventure Educ Outdoor Learn. 2016;16(3):239–55.

[77] Waite S, Husain F, Scandone B, Forsyth E, Piggott H. ‘It’s not for people like (them)’: structural and cultural barriers to children and young people engaging with nature outside schooling. J Adventure Educ Outdoor Learn. 2023;23(1):54–73.

[78] Aggio D, Smith L, Fisher A, Hamer M. Mothers’ perceived proximity to green space is associated with TV viewing time in children: The Growing Up in Scotland study. Prev Med. 2015;70:46–9. 25434736 10.1016/j.ypmed.2014.11.018 PMC4295935

[79] Dunton GF, Kawabata K, Intille S, Wolch J, Pentz MA. Assessing the social and physical contexts of children’s leisure-time physical activity: an ecological momentary assessment study. Am J Health Promot. 2012;26(3):135–42. 22208410 10.4278/ajhp.100211-QUAN-43

[80] Keith RJ, Given LM, Martin JM, Hochuli DF. Environmental self-identity partially mediates the effects of exposure and connection to nature on urban children’s conservation behaviours. Curr Res Ecol Soc Psychol. 2022;3:100066.

[81] Lu C, Shen T, Huang G, Corpeleijn E. Environmental correlates of sedentary behaviors and physical activity in Chinese preschool children: a cross-sectional study. J Sport Health Sci. 2022;11(5):620–9. 10.1016/j.jshs.2020.02.010 PMC9532589 32360638

[82] Rosen ML, Rodman AM, Kasparek SW, Mayes M, Freeman MM, Lengua LJ, et al. Promoting youth mental health during the COVID-19 pandemic: a longitudinal study. PLoS One. 2021;16(8):e0255294. 34379656 10.1371/journal.pone.0255294 PMC8357139

[83] Schoeppe S, Vandelanotte C, Bere E, Lien N, Verloigne M, Kovács É, et al. The influence of parental modelling on children’s physical activity and screen time: does it differ by gender? Eur J Pub Health. 2016;27(1):152–7. 10.1093/eurpub/ckw182 28177458

[84] Sheldrake R, Reiss MJ. Primary children’s views about appreciating, supporting, and learning about nature. J Biol Educ. 2023;57(2):401–21.

[85] Shyleyko R, Luca P, Jackman M, Ho J. Reported barriers to physical activity and the role of built environment among overweight and obese youth attending a Canadian pediatric weight management clinic. Prev Med Rep. 2023;36:102488. 38116277 10.1016/j.pmedr.2023.102488 PMC10728311

[86] AktasArnas Y, Saribas S. An investigation of pre-school children’s and their parents’ outdoor play experiences = Okul öncesi dönem çocuklari ve ebeveynlerinin açik hava oyun deneyimlerinin incelenmesi. Pegem J Educ Instruction. 2020;10(2):373–97.

[87] Ahmetoglu E. The contributions of familial and environmental factors to children’s connection with nature and outdoor activities. Early Child Dev Care. 2019;189(2):233–43.

[88] Zhang Y, Zhang Y, van Dijk T, Yang Y. Green place rather than green space as a health determinant: a 20-year scoping review. Environ Res. 2022;214:113812. 10.1016/j.envres.2022.113812 35970380

[89] Michaelson V, King N, Janssen I, Lawal S, Pickett W. Electronic screen technology use and connection to nature in Canadian adolescents: a mixed methods study. Can J Public Health. 2020;111(4):502–14. 32026342 10.17269/s41997-019-00289-y PMC7438459

[90] Yalçin SS, Tezol Ö, Çaylan N, EratNergiz M, Yildiz D, Çiçek Ş, et al. Evaluation of problematic screen exposure in pre-schoolers using a unique tool called “seven-in-seven screen exposure questionnaire”: cross-sectional study. BMC Pediatr. 2021;21(1):472. 34696746 10.1186/s12887-021-02939-y PMC8546938

[91] Halpin S, Mitchell AE, Baker S, Morawska A. Parenting and child behaviour barriers to managing screen time with young children. J Child Fam Stud. 2021;30(3):824.

[92] Cameron-Faulkner T, Melville J, Gattis M. Responding to nature: Natural environments improve parent-child communication. J Environ Psychol. 2018;59:9–15.

[93] Izenstark D, Ravindran N, Rodriguez S, Devine N. The affective and conversational benefits of a walk in nature among mother-daughter dyads. Appl Psychol Health Well Being. 2021;13(2):299–316. 33755327 10.1111/aphw.12250

[94] Izenstark D, Ebata AT. The effects of the natural environment on attention and family cohesion: an experimental study. Child Youth Environ. 2017;27(2):93–109.

[95] Weeland J, Moens MA, Beute F, Assink M, Staaks JPC, Overbeek G. A dose of nature: two three-level meta-analyses of the beneficial effects of exposure to nature on children’s self-regulation. J Environ Psychol. 2019;65:101326.

[96] Richardson M, McEwan K, Maratos F, Sheffield D. Joy and calm: How an evolutionary functional model of affect regulation informs positive emotions in nature. Evol Psychol Sci. 2016;2:308–20.

[97] McCree M, Cutting R, Sherwin D. The Hare and the Tortoise go to Forest School: taking the scenic route to academic attainment via emotional wellbeing outdoors. Early Child Dev Care. 2018;188(7):980–96.

[98] Taylor AF, Butts-Wilmsmeyer C. Self-regulation gains in kindergarten related to frequency of green schoolyard use. J Environ Psychol. 2020;70:101440.

[99] Barnes C, Harvey C, Holland F, Wall S. Development and testing of the Nature Connectedness Parental Self-Efficacy (NCPSE) scale. Urban Forest Urban Green. 2021;65:127343.

[100] Albanese AM, Russo GR, Geller PA. The role of parental self-efficacy in parent and child well-being: a systematic review of associated outcomes. Child Care Health Dev. 2019;45(3):333–63. 30870584 10.1111/cch.12661

[101] Jiang B, Schmillen R, Sullivan WC. How to waste a break: Using portable electronic devices substantially counteracts attention enhancement effects of green spaces. Environ Behav. 2019;51(9–10):1133–60.

[102] Hitron T, David I, Ofer N, Grishko A, Wald IY, Erel H, Zuckerman O. Digital Outdoor play: Benefits and risks from an interaction design perspective. In: Proceedings of the 2018 CHI conference on Human Factors in Computing Systems; 2018. p. 1–13.

[103] Price E, Maguire S, Firth C, Lumber R, Richardson M, Young R. Factors associated with nature connectedness in school-aged children. Curr Res Ecol Soc Psychol. 2022;3:100037.

[104] Richardson M, Hussain Z, Griffiths MD. Problematic smartphone use, nature connectedness, and anxiety. J Behav Addict. 2018;7(1):109–16. 29415553 10.1556/2006.7.2018.10 PMC6035021

[105] Vella K, Johnson D, Cheng VWS, Davenport T, Mitchell J, Klarkowski M, et al. A sense of belonging: Pokémon GO and social connectedness. Games Culture. 2019;14(6):583–603.

[106] del Borja P-C, Francisco P, Phil P, Chris L, Michael N, Kylie DH, et al. Joint physical-activity/screen-time trajectories during early childhood: socio-demographic predictors and consequences on health-related quality-of-life and socio-emotional outcomes. Int J Behav Nutr Phys Act. 2019;16(1):1–13. 31286983 10.1186/s12966-019-0816-3 PMC6615223

[107] Trott M, Driscoll R, Iraldo E, Pardhan S. Changes and correlates of screen time in adults and children during the COVID-19 pandemic: a systematic review and meta-analysis. eClinicalMedicine. 2022;48:101452. 35615691 10.1016/j.eclinm.2022.101452 PMC9122783

[108] Mallawaarachchi SR, Tieppo A, Hooley M, Horwood S. Persuasive design-related motivators, ability factors and prompts in early childhood apps: a content analysis. Comput Hum Behav. 2023;139:107492.

[109] The Royal Children’s Hospital. National Child Health Poll. Melbourne (Australia): The Royal Children’s Hospital; 2021.

